# Interactions between Hyaluronan and Its Receptors (CD44, RHAMM) Regulate the Activities of Inflammation and Cancer

**DOI:** 10.3389/fimmu.2015.00201

**Published:** 2015-05-06

**Authors:** Suniti Misra, Vincent C. Hascall, Roger R. Markwald, Shibnath Ghatak

**Affiliations:** ^1^Department of Regenerative Medicine and Cell Biology, Medical University of South Carolina, Charleston, SC, USA; ^2^Department of Biomedical Engineering, Cleveland Clinic, Cleveland, Ohio, OH, USA

**Keywords:** hyaluronan, CD44, RHAMM, inflammation, cancer

## Abstract

The glycosaminoglycan hyaluronan (HA), a major component of extracellular matrices, and cell surface receptors of HA have been proposed to have pivotal roles in cell proliferation, migration, and invasion, which are necessary for inflammation and cancer progression. CD44 and receptor for HA-mediated motility (RHAMM) are the two main HA-receptors whose biological functions in human and murine inflammations and tumor cells have been investigated comprehensively. HA was initially considered to be only an inert component of connective tissues, but is now known as a “dynamic” molecule with a constant turnover in many tissues through rapid metabolism that involves HA molecules of various sizes: high molecular weight HA (HMW HA), low molecular weight HA, and oligosaccharides. The intracellular signaling pathways initiated by HA interactions with CD44 and RHAMM that lead to inflammatory and tumorigenic responses are complex. Interestingly, these molecules have dual functions in inflammations and tumorigenesis. For example, the presence of CD44 is involved in initiation of arthritis, while the absence of CD44 by genetic deletion in an arthritis mouse model increases rather than decreases disease severity. Similar dual functions of CD44 exist in initiation and progression of cancer. RHAMM overexpression is most commonly linked to cancer progression, whereas loss of RHAMM is associated with malignant peripheral nerve sheath tumor growth. HA may similarly perform dual functions. An abundance of HMW HA can promote malignant cell proliferation and development of cancer, whereas antagonists to HA-CD44 signaling inhibit tumor cell growth *in vitro* and *in vivo* by interfering with HMW HA-CD44 interaction. This review describes the roles of HA interactions with CD44 and RHAMM in inflammatory responses and tumor development/progression, and how therapeutic strategies that block these key inflammatory/tumorigenic processes may be developed in rodent and human diseases.

## Introduction

The interplay between cells and with components in the extracellular matrix (ECM) of the microenvironment is tightly regulated during normal physiological processes of tissues and organs (1–3). During inflammation and tumorigenesis, cellular communications are dramatically lost with subsequent extensive remodeling of cellular and molecular composition of the tumor microenvironment, which supports inflammation, cancer cell proliferation and migration/motility, invasion, and metastasis. Carcinogenesis is characterized by an inflammatory response where biologically active ECM fragments regulate tissue injury/remodeling. Notably, the biosynthesis and degradation of the glycosaminoglycan (GAG) hyaluronan (HA), a major component in ECMs, is associated with the rapid matrix remodeling that occurs during embryonic morphogenesis, inflammation, and tumorigenesis (4–7).

**Figure 1:**
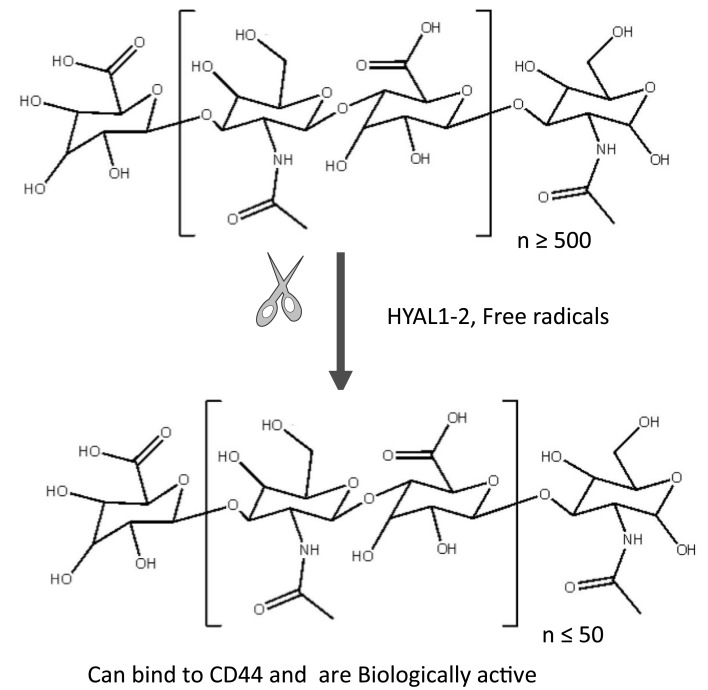
**Native polymeric HA and degraded HA fragments**. High molecular weight (HMW ≥ 500 disaccharide units; [β-1, 4-GlcUA-β-1, 3-GlcNAc]n synthesized in the normal homeostatic condition is degraded by free radicals and hyaluronidases (HYAL1-2) during inflammation/tumorigenesis when tissue injury occurs. These fragments are ≤50 disaccharide units. As a result, the fragments of different molecular weights have different biological functions. For example, intermediate fragments (30–500 kDa) can stimulate cell proliferation while smaller fragments <50 kDa promote cell migration. HA oligosaccharides down to three disaccharides can still bind to CD44.

**Figure 2:**
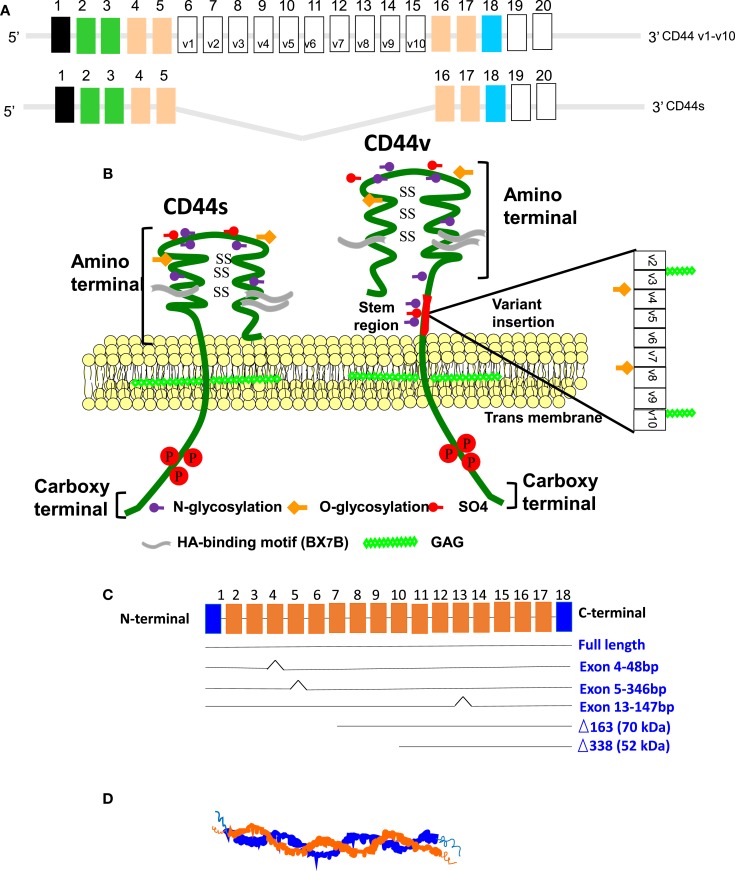
**(A)** Mouse CD44 and RHAMM gene/protein structures in mice. Model structure of alternative splicing in CD44. CD44 pre-mRNA is encoded by 20 exons in mouse and 19 exons in human being. The common standard CD44s (hematopoietic) form contains no extra exons, and the protein has a serine motif encoded in exon 5 that can initiate synthesis of a chondroitin sulfate or dermatan sulfate chain. Alternative splicing of CD44 predominantly involves variable insertion of 10 extra exons with combinations of exons 6–15 and spliced in v1–v10 into the stem region, of which v3 encodes a substitution site for a heparan sulfate chain. Variable numbers of the v exons can be spliced in epithelial cells, endothelial cells, and inflammatory monocytes and also are upregulated commonly on neoplastic transformation depending on the tissue. **(B)** Model structure of alternatively spliced CD44 proteins. The CD44 protein is composed of an extracellular N-terminal domain, a stem region in the extracellular domain close to the transmembrane region, where the variant exon products (red/violet circles) are inserted, the transmembrane region, and the carboxyl terminal cytoplasmic tail. There are multiple sites for *N*-glycosylation (purple circles) and *O*-glycosylation (orange circles), and a sulfation domain. The N-terminal portion contains highly conserved disulfide bonds as well as 2 BX7B motifs, both of which are essential for HA binding. CD44 is subjected to extensive glycosylation, sulfation, and attachment of GAGs that contribute to regulation of the HA-binding activity. The C-terminal cytoplasmic tail contains several phosphorylation sites that regulate the interaction of CD44 with the cytoskeletal linker proteins, as well as with SRC kinases. **(C)** RHAMM exon structure. The full-length protein (85 kDa in human beings) is largely associated with microtubule formation during the cell cycle progression. Three isoforms are generated by alternative splicing of exon 4, 5, or 13. Loss of exon 4 disrupts association with microtubules and results in the appearance of RHAMM in the cell nucleus. N-terminal truncations that may be generated by a posttranslational mechanism are constitutively present in some aggressive breast cancer cell lines and tumors. These accumulate in the nucleus and on the cell surface. **(D)** The secondary structure of RHAMM. RHAMM can self-associate to form random coiled coils (132).

Hyaluronan is a non-sulfated, linear GAG composed of repeating disaccharides of (β, 1–4)-glucuronic acid (GlcUA) and (β, 1-3)-*N*-acetyl glucosamine (GlcNAc) (MW ~ 400 Da) (Figure 1). Native HA in most tissues has a high molecular mass of 1–10 million Da with extended molecular lengths of 2–20 μm (8–11). HA has crucial roles in structuring tissue architecture, in cell motility, in cell adhesion, and in proliferation processes (12, 13). These cellular events are mediated mainly through two major signal-transducing cell surface HA-receptors, CD44 (14–19) and the receptor for HA-mediated motility (RHAMM) designated as CD168 (20) (Figure 2), which was first described by Turley (21, 22) as a soluble HA-binding protein released by sub-confluent migrating cells (23). HA is the principal ligand of CD44 (16), and alternative splicing and differential glycosylation produce multiple structural and functional versions of CD44 that are responsible for proinflammatory activities, including cell–cell and cell–matrix interactions (6, 24–26). The CD44 ectodomain includes an amino-terminal domain that contains a HA-binding “link module” motif related to those in the HA-binding proteoglycans and the link proteins (27). Like CD44, RHAMM is alternatively spliced, and variant forms of RHAMM are found both on cell surfaces and inside the cells (28, 29). However, they do not have the link module domain. They have a BX7B motif that also can bind to HA, where “B” represents arginine or lysine, and “X” represents any non-acidic amino acid (30). Studies indicate that CD44-mediated cell migration during inflammation, wound healing, and tumorigenesis can require surface expression of RHAMM. However, the mechanism of cooperativity between RHAMM and CD44 is not clearly understood. In this review, we discuss the nature of such interactions and the important therapeutics that can target CD44 and RHAMM in inflammation and cancer.

## HA in Inflammation and Cancer

### Biology of HA

Hyaluronan is a ubiquitous component of tissue ECM found in a native homeostatic form within hydrated tissues such as the vitreous of the eye, articular cartilage, synovial fluid, lymphatics, and skin. HA is present in interstitial collagenous matrices and increases viscosity and hydration. This impedes fluid flow through matrices by forming a barrier (31). HA is found in pericellular matrices attached to the HA-synthesizing enzymes or its receptors and is also present in intracellular degradation compartments (6, 7, 32–37). HA differs from the other GAGs: it is not synthesized on a core protein as a proteoglycan in the Golgi. Instead it is synthesized by single transmembrane HA synthases (HAS1, HAS2, or HAS3) at the inner face of the plasma membrane. The cytoplasmic active HAS sites alternately add the UDP-GlcUA and UDP-GlcNAc substrates to the reducing, UDP-anchored elongating HA molecule that is being systematically extruded through the plasma membrane into the ECM to form the very long, unmodified HA macromolecules without any sulfated or epimerized uronic acid residues (23, 38). The HAS isozymes (HAS1, HAS2, HAS3) differ from each other in their catalytic activities as well as in the sizes of the synthesized HA. HAS1 and HAS2 polymerize long high molecular weight (HMW) chains while HAS3 produces predominantly shorter low molecular weight (LMW) chains (<300 kDa), and HAS3 appears to be more active than HAS1 and HAS2 (38–40).

While its structure is simple, HA is an extraordinarily versatile macromolecule. Its biophysical properties provide HA with functions that influence the hydration and biomechanical properties of different tissues, especially those of the vitreous humor in the eye, the synovial joint fluid, and the dermis (41). In addition, HA also interacts with extracellular macromolecules and HA-binding proteoglycans, including versican and aggrecan, which are important in the assembly of ECMs and of pericellular glycocalyces that can act as protective cellular barriers and are essential for the assembly and structure of many tissues (41–44). For example, increased levels of aggrecan immobilized on HA in collagen networks resists the variable compressive loads essential for the physical properties of cartilages (45).

**Figure 3:**
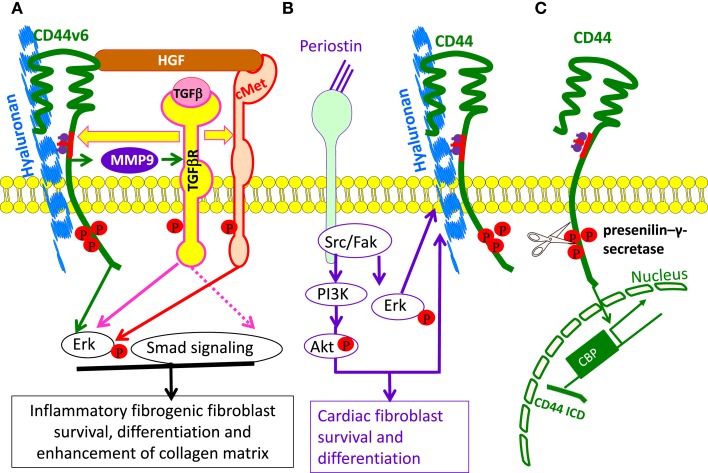
**The involvement of HA and CD44 in cell survival and differentiation**. **(A)** Model for the involvement of CD44v6 and Met due to autocrine TGF β1 signaling in lung fibrogenic fibroblasts. The repetitive lung injury in pulmonary fibrosis results in overexpression of TGFβ1 and TGFβ1-induced autocrine signaling that induces a sustained expression of CD44v6 and its co-receptor c-Met. This activates fibrogenic lung fibroblasts with subsequent increased collagen matrix synthesis. Therefore, TGF β1-induced CD44v6 and Met can have a crucial role for the sustained fibrogenic activation of lung fibroblasts. The CD44-phosphorylated ERM complex initiates activation of transforming growth factor-β receptor 1 and 2 (TGFβRI and II) and the downstream SMAD signaling complex, which contribute to fibrosis. **(B)** Model for involvement of periostin in HA-CD44-mediated cell survival and differentiation. Matricellular protein [periostin (PN)] binding to β1 or β3-integrin activates FAK, which activates downstream MAPK/Erk and PI3K/Akt to regulate cardiac valve cell growth, survival, differentiation into fibroblasts, and matrix organization (maturation). PN binding to β3-integrin also activates Has2 mRNA expression, Has2 phosphorylation, and HA synthesis. The interaction of HA with CD44, may, in turn, amplify the downstream effects of PN on heart valve cushion cell differentiation/maturation processes. **(C)** Cleavage of the extracellular domain is accompanied by the cleavage of the intracellular domain (ICD) by the presenilin-γ-secretase complex. The CD44 ICD acts together with CBP or p300 as a transcription factor and promotes CD44 transcription and extracellular matrix production.

Successful morphogenesis also relies on physical properties of HA as well as on signaling events triggered by HA-CD44 and/or HA-RHAMM interactions. During embryogenesis, HA promotes proliferation and migration of undifferentiated stem cells to sites of organ development (26). Importantly, Has2 null mice fail to synthesize HA during cardiac cushion development, and the endothelial cushion cells do not undergo mesenchymal transformation (EMT) and cannot form the underlying connective tissue, which leads to midgestational death (5, 46). Recent studies also indicate that the matricellular protein periostin binding to the integrins activates the HA synthesis and HA-mediated Akt/PKB and focal adhesion kinase (FAK)/Erk signaling pathways, which by feedback loop, further sustains Has2 expression for cell survival, and importantly, differentiation of embryonic cardiac mitral valve cells (47) (Figure 3B).

Hyaluronan turnover is important for the maintenance of tissue homeostasis, and ~30% of HA is replaced by newly formed HA per 24 h (48). Removal of HA occurs by endocytic uptake within the tissue, especially in lymph nodes and liver. Importantly, the presence of reactive oxygen species (ROS) enhances HA turnover (49–51). Internalization and degradation of HA is triggered by its binding to CD44 (52) and/or lymphatic vessel endothelial receptor-1 (LYVE-1), which is expressed in lymphatic endothelium (53). HA is degraded into monosaccharides by three enzymatic reactions. Hyaluronidases (HYALs) degrade HA to oligosaccharides, which are then digested into GlcUA and GlcNAc by β-d-glucuronidase and β*-N*-acetyl-d-hexosaminidase (54, 55). In human beings, there are six HYAL genes, HYAL1-4, HYAL-P1, and sperm-specific PH-20 (54, 56–58). These enzymes can have different catalytic profiles. For example; HYAL1 degrades HA into oligosaccharides while HYAL2 degrades HMW HA into fragments of intermediate size (~20 kDa). PH-20 is anchored to the sperm acrosomal membrane through a glycosyl-phosphatidylinositol (GPI) moiety, which promotes penetration of spermatozoa through the HA matrix in cumulus-oocyte complexes for fertilization (54, 59). In recent years, recombinant human PH20 devoid of the GPI anchor has been prepared (60) and tested for increasing drug penetration in skin and pancreatic tumors in an animal model (61).

As mentioned earlier, under physiological conditions, HA exists as a HMW polymer >10^6^ Da providing structural frameworks for cells. Such HMW HA functions as an extracellular molecule transmitting signals and regulates a variety of cell behaviors, including cell adhesion, motility, and growth (6, 8–11, 37). HA chains up to 20 MDa are involved in ovulation, embryogenesis, wound repair, and tissue regeneration (62). In malignant cells, HA polysaccharides (>10^6 ^Da) are abundant. HA levels can be increased around tumor cells themselves or within the tumor stroma to maintain normal cellular functions of the tumor cells. Because of the close association of high HA levels with malignancy in many tumor types (37) considerable experimental evidence implicates HA and its main receptor CD44 variants in tumor progression both in cell and animal models (6, 25, 37, 63–69). HMW HA induce activation of tyrosine kinase in endothelial cells and Ras-transformed fibroblasts at lower level (70, 71) compared to fragmented HA (71). However, the ability of the naked mole rat to synthesize high molecular mass HA (five times larger than human HA) is correlated to the cancer resistance and longevity of naked mole rat (72), but this animal is a rare species. A recent study established that reducing HAS levels using antisense techniques suppresses tumor growth, but showed that extremely high HA levels also inhibit tumor growth, possibly by suppressing cell–cell interactions, or ligand access to cell surface receptors (73). Remarkably, following tissue injury, HA and its degraded fragments <5 × 10^5^ Da size accumulate. Such smaller size HA was shown to be crucial for the production of cytokine and chemokine for differentiation/activation of the macrophage (74–76). HA of size <5 × 10^5^ Da (~250,000 Da) induce inflammatory genes with renal tubular epithelial cells (77), T-24 carcinoma cells (78), and eosinophils (79). Similarly, HA fragments of size 8–16 disaccharides induce angiogenesis in a chick corneal assay, whereas the native HA molecules do not (80–82). Similarly, smaller HA fragments in the 6–20 kDa size range induce inflammatory gene expression in dendritic cells (83). Studies have shown that HA of 40–400 kDa interact with HA-receptors to activate the NFκB-mediated gene expression for endocytosis (84). Under certain conditions, HA fragments (20–200 kDa size) function as endogenous danger signals, while even smaller HA fragments (~2,500 kDa) can ameliorate these effects in cancer (37, 85, 86). Thus, generation of HA fragments by HYALs (87) or by free radicals (88) during inflammation/tumorigenesis/tissue injury send the signals to the injured host that normal HA homeostasis is disturbed due to activation of different signal transduction pathways. The biological function of various sizes of HA is presented in Table 1.

**Table 1:** **Various sizes of HA and their function in health and disease**.

Sizes (kDa, or disachharide)	Function of HA	References
HA size > 10^6 ^Da	Space-filling, providing structural frameworks for cells, functions as an extracellular molecule transmitting signals, and regulates a variety of cell behaviors, including cell adhesion, motility, and growth	(6, 8–11, 37)
HA size > 10^6 ^Da	Rapid matrix remodeling that occurs during embryonic morphogenesis, as well as tumorigenesis	(4–7, 27, 37, 63–69)
HA size > ~20 MDa	Ovulation, embryogenesis, wound repair, and tissue regeneration	(62)
HA size > 10^6 ^Da	Tyrosine kinase in endothelial cells and Ras-transformed fibroblasts at lower level	(70, 71)
Extremely HMW HA (five times larger than human HA)	Cancer resistance and longevity of naked mole rat	(72, 73)
Excessive HMW HA production	Inhibit tumor growth, possibly by suppressing cell–cell interactions, or ligand access to cell surface receptors	
HA size <5 × I0^5^ Da	Cytokine and chemokine production for differentiation/activation of the macrophage	(74–76)
HA size ~250,000	Induces inflammatory genes in renal tubular epithelial cells, T-24 carcinoma cells, and in eosinophils	(77–79)
HA size 8–16 disaccharides (~3200–6400 Da)	Induce angiogenesis in a chick corneal assay whereas the native HA molecules do not	(80–82)
HA size ~6–20 kDa	Induce inflammatory gene expression in dendritic cells	(83)
HA of 40–400 kDa	NFkB-mediated gene expression for endocytosis	(84)
HA size ~2,500 Da	Upregulation of PTEN in tumor cells, and inhibit anchorage-independent growth as well as xenograft tumor growth	(37, 86)

Both physiological and pathological functions of HA are mediated by molecular interactions with CD44 and RHAMM and other HA-binding proteins (42). HA-rich glycocalyces anchored on cell surfaces by CD44 and RHAMM can activate intracellular signaling pathways (6, 42, 89), which can induce gene expression related to cell–cell adhesion, cell spatial orientation and trafficking, cell growth and differentiation, inflammation (11), wound healing and tissue remodeling (11, 80, 90), tissue morphogenesis (11, 91) matrix organization, and many inflammatory pathologies (6, 7, 19, 33–37, 92–100).

### HA in inflammation

The cell microenvironment has an important role in controlling inflammation. Prolonged inflammation leads to an influx of inflammatory cells, and it is increasingly clear that ECM degradation products are not only the result of inflammation but also can perpetuate inflammatory processes. LMW HA interactions with CD44 are associated with certain pathological conditions, including inflammation, cancer, and induction of angiogenesis (23, 33, 101–103).

Hyaluronan chains can be organized into ECM structures by association with binding proteins. For example, HA cross-linking is essential to stabilize the cumulus-oocyte complex and is needed for successful ovulation and fertilization. The same mechanisms are likely to occur at sites of inflammation, where HA synthesis is upregulated (104, 105). Importantly, HA complexes of indeterminate sizes originate from the surface of multiple cells and can be coalesced into cable-like structures or HA fibrils at sites of tissue inflammation, viral infection, endoplasmic reticulum (ER) stress, and hyperglycemia. Monocytes/macrophages adhere to these HA inflammatory matrices and fragment them by a mechanism that involves cell surface HYAL2 and CD44, which forms a cap on the surface while a portion of the HA matrix is internalized. Therefore, HA cross-linking represents an important new pathway in the regulation of inflammatory processes (33, 105–110).

One of the functions of HMW HA is to maintain water homeostasis and matrix structure (111). However, during inflammation, there is increased breakdown of HMW HA by HYALs (62), resulting in the accumulation of LMW forms that have different functions than their HMW precursors (101, 112) (see Figure 1). LMW HA is a potent activator of macrophages and airway epithelial cells (113–115). Specifically, LMW HA (~200 kDa) can induce the expression of proinflammatory genes, such as macrophage inflammatory protein (MIP), keratinocyte chemoattractant (KC), macrophage chemoattractant protein-1 (MCP-1), and IFN induced protein-10, as well as cytokines, such as IL-8, IL-12, and TNF-α (76, 101, 112, 115–117). HA-stimulation of macrophages is associated with matrix-modifying enzymes (MMEs), inducible nitric oxide synthase (iNOS), and plasminogen activator inhibitor (74, 116, 118). In addition, HA is also depolymerized by ROS, and the HA fragments are implicated in chronic inflammation (119). These HA-induced inflammatory mediators can enhance the inflammatory response that has already been set in motion, sending the system into a positive-feedback loop where inflammation promotes further inflammation, which, if unchecked, may eventually lead to fibrosis.

### HA in cancer

There is a complex cross-talk between cancer cells and their microenvironment. Strong evidence indicates that the tumor microenvironment can regulate the capacity of tumors to grow and metastasize (120). HA not only provides a cellular support and hydrophilic matrix but also regulates cell–cell adhesion, cell migration, growth, and differentiation (11). These properties make it a suitable candidate for involvement in pathological processes such as cancer. Furthermore, by forming pericellular coats, HA can protect tumor cells from immune attack (43, 44). Several tumor cells produce increased amounts of HA or induce the production of HA by the release of growth factors and cytokines. Such signals can regulate both HAS1, 3 and HYAL1. Similarly, ROS-induced fragmented HA also contributes to overproduction of HA (54). Likewise, both tumor cells and stromal cells express HAS isoforms and produce HA ECM, which then accumulates in tumor parenchyma and in the peritumor stromal tissues, which contributes to metastatic spreading (121–125). Moreover, HA overproduction in tumor cells may induce EMT-like epithelial changes of cancer cells toward a migratory fibroblastic phenotype (126). HA-rich ECM may also mediate the recruitment of mesenchymal stem cells, which are progenitors of tumor-associated fibroblasts (127).

In addition, HAS1–3 synthesize HA of dissimilar sizes, which are linked to specific HA functions in wound injury (37, 125, 128–131) as seen in keratinocyte wound repairs, and during mechanical injury in mesothelial cells (73, 132–136). Increased production of HA in non-malignant cells impairs contact inhibition of cell growth and migration (137). Likewise, forced expression of HAS2 and HAS3 genes results in HA overproduction, which enhances the tumor forming ability of fibrosarcomas and melanoma cells (67, 68) while abrogation of HAS2 blocks xenograft prostate tumor growth (69). Transgenic mouse models have shown that tumor-derived HA induces stromal reactions with subsequent promotion of tumor angiogenesis and lymphatic penetration within intratumoral stromal compartments (126). In addition, HA-rich tumor-associated micro-environments also accelerate the recruitment of inflammatory cells and the production of cytokines and chemokines, and the HA fragments generated by the degradation of HA can stimulate tumor growth and angiogenesis (82). Thus, targeting HA-tumor cell receptor interactions may identify promising therapeutic approaches in cancer treatment. In addition to interaction with cell surface receptors that initiate signaling pathways that impair vascular function (138), HA can increase interstitial fluid pressure and form a physical barrier against movement of small molecule therapeutics especially in pancreatic ductal adenocarcinomas (61). Thus, depolymerizing HA with HYAL may improve vascular function and ease movement of therapeutics.

## Interaction of CD44 and RHAMM with HA

### Biology of CD44

CD44 is a broadly distributed cell surface glycoprotein found on hematopoietic cells, fibroblasts, and numerous tumor cells. It was first identified as gp85 (14) and was then shown to be a HA-receptor in placenta cells when their adherence to immobilized HA was inhibited by an anti-CD44 monoclonal antibody, by soluble HA, and by HYAL (16, 139–142). However, the presence of the amino-terminal HA-binding region of CD44 does not guarantee that cells expressing CD44 will bind HA. Indeed, most CD44-expressing cells taken from normal animals, as well as from CD44+ cell lines, do not bind HA. Binding of CD44 to HA is cell specific and depends on the activation state of CD44 (143). CD44 has seven extracellular domains, a transmembrane domain, and a cytoplasmic domain (Figure 2) (144). The extracellular structure has two regions (amino acids 21–45 and amino acids 144–167) that contain clusters of conserved basic residues that have been implicated in HA binding, the BX7B motif. This motif, which is found in other HA-binding proteins, including RHAMM, is present as a single copy in the first of these regions, and as an overlapping pair in the second region (Figure 2). The pairs of intra-molecular disulfide bonds are also crucial for HA-binding activity. The HA-binding domain located in the amino-terminal region is present in all isoforms (145). The membrane proximal region is less well conserved and contains the insertion site for the variant exon domains. The transmembrane and C-terminal cytoplasmic domains are highly conserved (142, 146, 147) (Figure 2).

CD44 is encoded by a single gene. Due to alternative splicing, multiple forms of CD44v are generated that are further modified by N- and O-linked glycosylation. The smallest CD44 standard isoform (CD44s) lacks variant exons, contains an N-terminal signal sequence (exon 1), a link module that binds to HA (exons 2 and 3), a stem region (exons 4, 5, 16, and 17), a single-pass transmembrane domain (exon 18), and a cytoplasmic domain (exon 20). In all forms of CD44 cDNAs, exon 19 is spliced out so that the transmembrane domain encoded by exon 18 is followed by the cytoplasmic domain encoded by exon 20, producing the 73 amino acid cytoplasmic domain. CD44s is found in most cells (6), whereas the isoforms that contain a variable number of exon insertions (v1–v10) at the proximal plasma membrane external region are expressed primarily on cells during inflammation and on tumor cells (24, 28, 65, 94) (Figure 2). More importantly, variants of CD44, specifically CD44v6, promote tumor progression and metastatic potential in lung, breast, and colon cancer (6, 24, 94, 95, 148, 149). Subsequently, several tumors, including colon cancer (150–154), Hodgkins lymphoma (155), gastric cancer (156), and melanoma (157), have been screened for CD44 isoforms, indicating that certain CD44 variants have crucial roles for tumor progression. HA and CD44s are present on the membrane of most vertebrate cells (65).

CD44 is a multifunctional receptor having diverse roles in cell–cell and cell–matrix interactions such as cell traffic, lymph node homing, prothymocyte homing, lymphocyte activation, cell aggregation, releasing chemokines and growth factors, and presenting them to traveling cells (158, 159). CD44 can be a proteoglycan with a potential chondroitin sulfate (CS) or dermatan sulfate (DS) substitution. Insertion of the v3 exon also includes the potential for heparan sulfate (HS) chain substitution (24), which can influence ligand binding and cell behavior by allowing CD44 to be a co-receptor for hepatocyte growth factor (HGF) with c-Met (160). The affinity of CD44 for these GAG substitutions depends on posttranslational modifications, such as oligosaccharide and GAG addition (161–163), and their subsequent functions depend on cell types and growth conditions (6, 24). These modifications can be altered by physiological stimuli, resulting in the induction of HA binding. In the immune system, HA binding can be induced in T cells upon antigen recognition (164) and upon monocyte activation by inflammatory stimuli (51, 52, 147). Unlike HAS2 deficient mice (19), CD44-null mice develop normally, indicating that CD44 is dispensable for development (53).

In addition to binding to HA, CD44 can interact with several ECM proteins, including fibronectin, collagens, growth factors, cytokines and chemokines, and matrix metalloproteinases (MMPs) (6, 24, 26). To contribute to pericellular proteolysis, CD44 facilitates activation of MMP-9 on the surface of carcinoma cells (165). CD44-mediated localization of MMP-9 on tumor cells can regulate tumor cell motility, growth factor activation, and survival mechanisms. It can also activate latent TGFβ and promote tumor invasion and angiogenesis (166) (Figure 3A). Tumor growth and metastasis is associated with increased levels of soluble CD44 (sol-CD44), which is detected in plasma from cancer patients (167), indicating increased proteolytic activity and matrix remodeling by CD44. Sol-CD44 is likely to affect cellular behavior by perturbing HA-CD44 interactions by acting as a sink for HA and may regulate cell migration by disrupting CD44-dependent cell–cell and cell–matrix adhesion. Cleavage of the extracellular domain of CD44 can be followed by subsequent proteolysis within the transmembrane domain and subsequent liberation of the CD44 intracellular domain (ICD) (168). The ICD can then translocate to the nucleus and promote transcription of CD44, providing a feedback mechanism for regulating CD44 expression (169) (Figure 3C).

Decades of research have shown that CD44 participates in major oncogenic signaling networks and in complexes with oncogenes that promote every aspect of tumor progression (6, 24). CD44 is extremely sensitive to changes in the microenvironment. For example, CD44 in breast cancer cells may act as a metastatic suppressor gene when influenced by ROS, as seen by decreased CD44 protein expression in the malignant and tumorigenic breast cancer alpha 5 cell line in a compensatory response to increased manganese superoxide dismutase (MnSOD) protein expression (170). Studies by Stoop et al. (171) showed that the cancer-initiating function in CD44-null mice was less severe, whereas the inflammatory functions were persistent in these mice suggesting again the possibility of a molecular redundancy in this model. Many of the contradictory findings published to date may be due to experimental and technical differences among studies. However, a picture has emerged suggesting that CD44 may function differently at different stages of cancer progression (172, 173). For example, mice with germline disruptions of CD44 display relatively mild phenotypes compared with mice in which tissue-specific CD44 function is disrupted at adult phases of development, or in later phases. This suggests that the absence of CD44 in early development and a loss of CD44 function late in development are tolerated differently (24). As CD44 is the major HA-receptor and a co-receptor for EGF, it was surprising to find that CD44-null mice had a mild phenotype. However, roles for this molecule in the immune system were revealed by a bacterial pneumonia model in which the null mice had enhanced edema and lung neutrophil accumulation (174). Therefore, CD44 appears to have a role in limiting inflammatory responses, which has also been shown in inflammation models (24).

Unlike CD44s, CD44v variants are only expressed on some epithelial cells during embryonic development, during lymphocyte maturation and activation, and in several types of carcinomas (175). In particular, upregulation of CD44v6 is an early event in carcinogenesis and requires adenomatous polyposis gene inactivation (153, 176, 177). We found that overexpression of HAS2 in pre-neoplastic Apc10.1 cells induces CD44v6 expression, and the intestinal/colon tumors of Apc1 Min/+ mice express CD44v6 at substantially higher levels compared to expression of CD44s (94). A considerable number of studies indicate that CD44 variant isoforms correlate with bad prognosis in patients with most human cancers (151, 178–184) except in neuroblastomas and prostate cancer (185, 186). CD44v6 is quite likely to be a suitable target for anticancer therapy because it is: (a) causally involved in metastasis of a rat pancreatic carcinoma (187); (b) redundantly correlates with the human tumors mentioned above; and (c) correlates with oncogenic functions in colorectal cancer (CRC) both *in vitro* and *in vivo* (6, 94, 178, 180, 181, 188).

### CD44 in inflammation

The role of CD44 in the immune system was first found when immune responses were examined using monoclonal CD44 antibodies (mAbs) in wild type mice. KM201 blocked HA-CD44 interaction, whereas IRAWB14 enhanced HA binding. IM7 induced the shedding of CD44 from the cell surface and induced neutrophil depletion (189–192), indicating that in addition to blocking HA-CD44 interaction, CD44 mAbs can also alter HA-independent functions, such as interactions of CD44 and E- or L-selectin. These approaches support a proinflammatory role for CD44 (193, 194).

Other studies show that leukocyte rolling on inflamed endothelium is not only mediated by the selectin molecules, but can also be mediated by the interaction of T cell CD44 with HA on activated microvascular endothelial cells (195, 196). Moreover, CD44 and HA can facilitate the recruitment of neutrophils to sites of inflammation in some instances (197–199). Reduced recruitment of CD44-null macrophages to atherosclerotic lesions (200) indicates the contribution of CD44 to monocyte/macrophage recruitment to inflammation sites. CD44-null mice also experienced reduced levels of cerebral ischemia injury, further supporting a proinflammatory role for CD44 (201, 202). Studies also revealed that treatment with anti-CD44 mAbs reduced the severity of arthritis in a collagen-induced mouse model for human rheumatoid arthritis (RA) (203–205) and reduced the diabetic activity in NOD nice (206). The decrease in disease severity was associated with the delayed access of donor lymphocytes into the RA joints of recipient animals (171, 207). In human RA, CD44v5, CD44v6, and CD44v10 have been detected in synovial fluid and serum of patients (208, 209). In an inflammatory bowel disease (IBD) model, expression of CD44v7 is crucial for colonic inflammation (210, 211). Furthermore, CD44v6 expression is associated with IBD severity in patients (212–214). Extensive HA matrix accumulates in bleomycin-induced lung fibrosis in CD44-null mice with persistent lung inflammation, extended chemokine production, impaired clearance of apoptotic lymphocytes, and death (215).

Our recent study showed that a feedback loop between CD44v6 and TGFβ1 augments the fibrogenic functions of lung fibroblasts in interstitial lung disease (92). In this study, we showed that TGFβ promotes c-Met expression and CD44v6 expression that is accompanied by the CD44v6-induced formation of α-SMA, increased cell proliferation and collagen production (Figure 3A). The CD44v6 signaling complex with TGFβRI and TGFβRII stimulates downstream SMAD signaling (Figure 3A). These findings provide clear evidence that TGFβI initiates the signaling cascade through CD44v6 toward differentiation of fibroblasts to myofibroblasts (92). They do not exclude a further contribution of CD44v6 by activating the TGFβ1 proform through associated MMPs (166, 216). Overall, these studies indicate the critical involvement of CD44 and its variants in a number of inflammatory situations. However, the specific role of CD44 depends on the model system and the disease.

### CD44 in cancer

Although studies *in vitro* indicate that the tumor promoting function of HA partly depends on its molecular weight (37, 86, 99, 217), and on its capacity to interact with other proteins (26, 218), many of the tumor promoting activities of HA could be explained by its interaction with CD44. There are three ways how CD44 can interact with HA.

#### CD44 Binds to Soluble Extracellular HA Molecules and ECM

CD44 proteins exist in three states with respect to HA binding: non-binding, non-binding unless activated by physiological stimuli, and constitutive binding (140, 219, 220). CD44 is endogenously expressed at low levels on various cell types in normal tissues (169), but it requires activation before it can bind to HA. Importantly, the minimal size of HA fragments binding to CD44 are six monosaccharide units (HA6). Thus, HMW HA in the ECM degraded by HYALs into smaller fragments can still bind to CD44 (221). Activated CD44 is overexpressed in solid tumors, but much less, or not at all on their non-tumorigenic counterparts. Adhesion of CD44 to HA induces upregulation of integrins that strengthen stem cell adhesion (222). Cross-talk between CD44 and CXCR4 signaling is a key role for HA and CD44 in CXCL12-dependent trans endothelial migration of stem cells (223). Tumor-derived cells express CD44 in a high-affinity state that is capable of binding and internalizing HA. Transitions from the inactive, low-affinity state to the active, high-affinity state by CD44 require posttranslational modifications, i.e., glycosylation in the extracellular domain and/or phosphorylation of specific serine residues in the cytoplasmic domain (26, 161) Such modulation of binding affinity of CD44 with HA is important for cellular migration that enables CD44 to be incorporated into the leading edge of the cells and lamellipodia (224).

CD44 can also react with other molecules, including collagen, fibronectin, osteopontin, growth factors (24), and MMPs in tumor cells (167, 225), but the functional roles of such interactions are less well known (24). Inhibiting cleavage of CD44 inhibits tumor cell migration on a HA substrate, suggesting that CD44 cleavage could release cells bound to a HA ECM (24). CD44 can also influence adhesion and de-adhesion to the ECM by regulating the pericellular HA matrix metabolism (226).

#### CD44 Interacts with Receptor Tyrosine Kinases for Anti-Apoptosis and Drug Resistance

Receptor tyrosine kinases (RTKs) are a subclass of cell surface growth factor receptors (GFRs) with an intrinsic, ligand-controlled tyrosine kinase activity (227). The cytoplasmic domains of RTKs contain catalytic kinase activity and phosphorylation motifs that on activated RTKs assemble many intracellular signaling molecules. Apart from their activation by the auto phosphorylation of cytoplasmic subunits of RTKs, they are also activated by their association with several proteins, which are known as co-receptors of RTKs. These co-receptors do not have kinase activity, but they modulate the kinase activity of RTKs.

**Figure 4:**
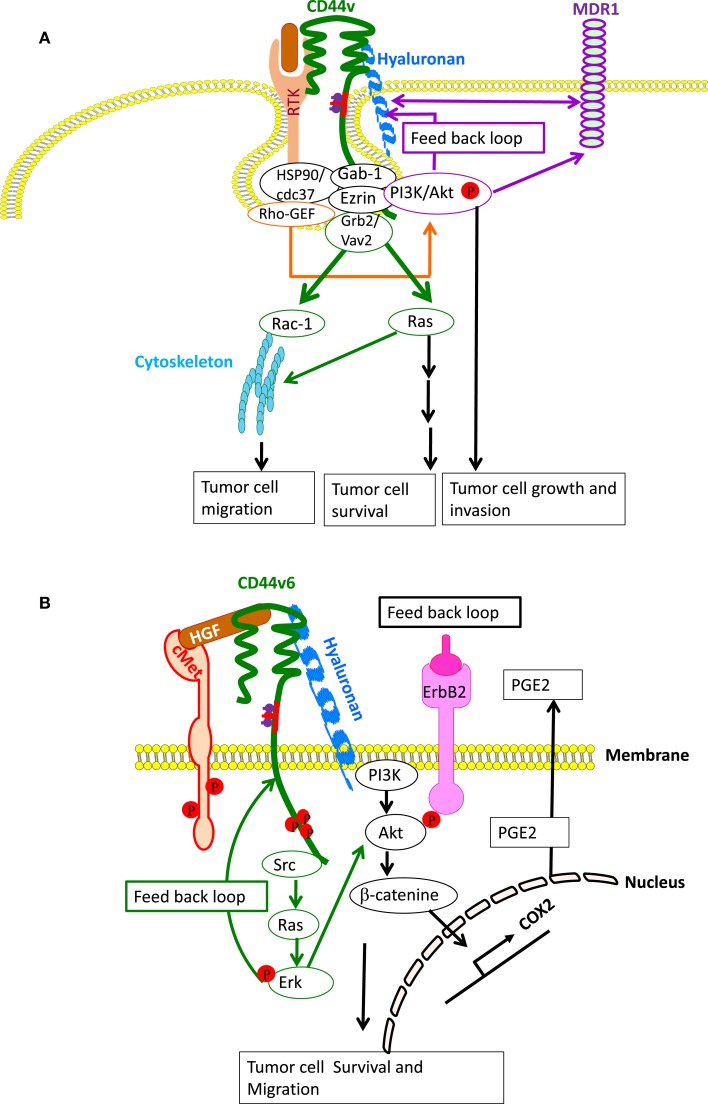
**CD44- and CD44v-induced RTK for apoptosis resistance**. **(A)** CD44-HA binding, accompanied by activation of CD44-associated SRC, ezrin phosphorylation, and PI3K activation leads to the lipid-raft-integrated assembly of a complex that includes heat shock protein 70 (HSP70), the co-chaperone CDC37, Rho-GEF, Grb2/VAV2, and Gab-1/PI3-kinase (PI3K), which promotes phosphorylation and activation of the receptor tyrosine kinases (RTKs), including ERBB2, ErbB3, EGFR, IGF1R-β, PDGFR-β, and c-MET. CD44-HA binding initiates cross-talk between RTKs, non-RTKs [SRC (Src)] and linker proteins. Studies have indicated that ErbB2 most likely complexes with CD44 via interactions with Grb2 and Vav2, whereas the interaction of PI3K and CD44 is mediated by Gab-1. PI3K activates Akt and downstream anti-apoptotic events, which contribute to drug resistance. However, HA and PI3K stimulate MDR1 expression, and the stimulatory effects of PI3K would be mainly due to its feedback stimulation of HA production by a positive feedback loop. Studies indicate that MDR1 is associated with CD44 in lipid microdomain and can be linked via CD44 with the actin cytoskeleton so that expression of both CD44 and MDR1 are concomitantly regulated. **(B)** Particularly, in colorectal cancer, the CD44–ERBB2 complex provides a strong apoptotic resistance through stimulation of cyclooxygenase 2 (COX2) transcription via PI3K and β-catenin. COX2-induced PGE2 stimulates HA synthesis and HA-CD44 signaling. CD44v6 also binds hepatocyte growth factor (HGF) and presents it to c-MET. Activation of MET and the downstream signaling cascades require sustained activation of CD44-associated phosphorylated ezrin, radixin, and moesin (ERM) and SRC (Src) signaling via the Ras-MAPK and the PI3K-Akt pathway. Ras-Erk pathway can augment CD44v6 synthesis through a feedback loop between CD44v6 and c-Met/Ras-Erk pathway.

HA-CD44 or HA-CD44v interaction has a general effect on activation of cell survival anti-apoptotic proteins, which is initiated through the association with RTK activation. In malignant colon, prostate, and breast carcinoma cells, HA-CD44 interaction activates multiple RTKs, including ERBB2, ErbB3, EGFR, IGF1R-β, PDGFR-β, and c-MET, as well as assembly of lipid-raft-integrated signaling complexes containing these activated RTKs, CD44, ezrin, PI3-kinase (PI3K) and the chaperone molecules HSP90 and CDC37, which strongly promotes apoptosis resistance in cancer cells (94, 96, 149, 228–231). Increased HA production, however, induces RTK activation and signaling complex assembly in phenotypically normal epithelial cells (96). These macromolecular signaling complexes of CD44 also contain RhoA-specific guanine nucleotide exchange factor (p115RhoGEF), which is upstream of Grb2-associated Ras and PI3-kinase (98) and VAV2, which regulates cytoskeletal reorganization through RAC1 activation (232) (Figure 4A). A blockade of the HA-CD44 interaction causes macromolecular lipid-raft-integrated complex disassembly and inactivation of RTKs in various cancer cells including breast, colon, and prostate cancer (6, 19, 93, 96, 188, 228–231). CD44 also associates with non-RTKs, such as SRC, which has a central role by linking various extracellular signals to crucial intracellular signaling pathways (233). Thus, the lipid-raft location of CD44 is of particular importance for the involvement of CD44 in cell motility and signal transduction and accounts for the CD44-HA-binding-initiated cross-talk between RTKs, non-RTKs, and linker proteins (234, 235). In colon cancer, the HA-CD44v6 interaction and recruitment of ERBB2 also induces the transcription of COX2 initiated downstream of CD44 through PI3K-Akt and β-catenin (93, 188). COX2 further strengthens apoptosis resistance and HA-CD44 interaction through prostaglandin E_2_ expression (93, 188) (Figure 4B). CD44v6 also initiates MET activation through HGF binding. This requires the cytoplasmic tail of CD44 and the interaction with ezrin, radixin, and moesin (ERM) proteins for activation of the Ras-MAPK pathway (236). In addition, CD44v6 binding to the ECM also activates the PI3K-Akt pathway (237, 238) (Figure 4B) and regulates *Met* transcription (239).

Hyaluronan-CD44 cross-linking regulates drug transporter expression (228, 240–242). In addition, a positive-feedback loop, involving HA, PI3K, and ErbB2, augments MDR1 expression and regulates drug resistance in breast cancer cells (228) (Figure 4A). MDR1 is associated with CD44 in lipid microdomain and can be linked via CD44 with the actin cytoskeleton so that expression of both CD44 and MDR1 are concomitantly regulated (240) (Figure 4A). HA-CD44 binding promotes protein kinase Cε (PKCε) activation, and this increases NANOG phosphorylation and translocation to the nucleus (243). These events initiate the upregulation of MDR1, and then CD44 can associate with and stabilize MDR1 gene expression (244). In contrast, pro-metastatic miR-373-520c downregulates CD44 (245) indicating that oncogenic miRNAs can regulate CD44 cell behavior in a specific way.

#### CD44 Can Act as Co-receptor

CD44v6 can function as co-receptor for RTKs and alter the activation of GFR kinases. The CD44v segments contain some specific posttranslational modifications that include HS on a site in exon v3, which can bind proteins such as fibroblast growth factor 2 (FGF2). Such a function is not confined to HS-modified CD44v3 isoforms. It can also be provided by CD44v6 isoforms, which act as co-receptors, i.e., CD44v6 contains a binding site for RTKs, HGF receptor c-Met, vascular endothelial growth factor (VEGF) receptor VEGFR-2, proteins involved in cancer, and in fibrosis (92, 103, 236, 246, 247). Thus, HA interactions with CD44v can have a central role in RTK activated cell pathways that promote tumorigenic functions, including cell survival, through the RTK activation and consequent MDR1 gene activation. Importantly, activation of signaling pathways initiated by HA-CD44v interactions in the tumor matrix can be inhibited by HA degradation, by inhibiting HA binding to CD44v with small HA fragments, by blocking the CD44v HA-binding site, or by CD44v knockdown (6, 94, 95, 248). Further, blockade of an individual RTK does not recapitulate all of the effects observed when HA-CD44v interaction is inhibited (149). HYAL, as well as low molecular mass HA oligosaccharides (oHAs), also improves drug efficacy (228, 241) and drug transporter expression (228, 242, 249) (Figure 8).

#### Binding of CD44 to Actin Cytoskeleton

CD44 and its associations with partner molecules, such as ERM proteins, participate in CD44-induced cell survival signaling, altered cell shape, and protein localization to the plasma membrane subdomains during cell migration (250). Ankyrin is involved in HA-CD44 dependent cell adhesion and motility. This involves binding of the N terminus of activated ERM to a motif between the transmembrane region and the ankyrin-binding site in the cytoplasmic domain of CD44, and subsequent binding of their carboxyl termini to filamentous actin (F-actin). This binds CD44 to cytoskeletal linker proteins, and this interaction is modulated by HA-CD44 interaction (251, 252). Overexpression of merlin, another member of the ERM family, inhibits subcutaneous growth of Tr6BC1 cells in immunocompromised Rag1 mice by negatively regulating CD44 function. In contrast, knocking down expression of endogenous merlin promoted tumor cell growth (253, 254).

### Biology of RHAMM

Receptor for HA-mediated motility, an acidic and coiled-coil protein designated as CD168 (20), was first identified as a component of the HA-receptor complex in the conditioned media of murine fibroblasts (21, 22). It is located intracellularly in the cytoplasm, in the nucleus, and on the cell surface (255). There is no link module domain in RHAMM, but it includes a HA-binding region through the BX7B motif on its COOH terminus (27). RHAMM lacks a transmembrane domain but is GPI-anchored to the cell membrane, where it can interact with CD44 and participate in many cell functions, including cell motility, wound healing, and modification of signal transduction of the Ras signaling cascade (28, 256–259). Surprisingly, RHAMM contains no signal peptide and is thought to be transported to the cell surface via unconventional transport mechanisms, where it associates with the cell surface via docking with HA synthase (260), and like CD44, it transduces signals that influence cell motility (28, 30).

### RHAMM signaling in inflammation and cancer

Extracellular RHAMM interacts with protein tyrosine kinase receptors (RTKs) and non-protein-TK receptors, including PDGFR (29), TGFβ receptor-1 (261), CD44 (259, 262), CD44-EGFR complexes (263, 264), bFGFR (265), and RON (266). These extracellular interactions can mediate motility necessary for inflammation through activation of ERK1/2/MAPK in the absence of intracellular RHAMM. Extracellular RHAMM can regulate cellular transformation and migration in an HA-dependent manner. It can bind to CD44 on mesenchymal cells in wounds, which is necessary for the sustained activation and nuclear translocation of activated ERK1, 2, and for cell migration with increased mesenchymal differentiation within wound sites (259, 267).

**Figure 5:**
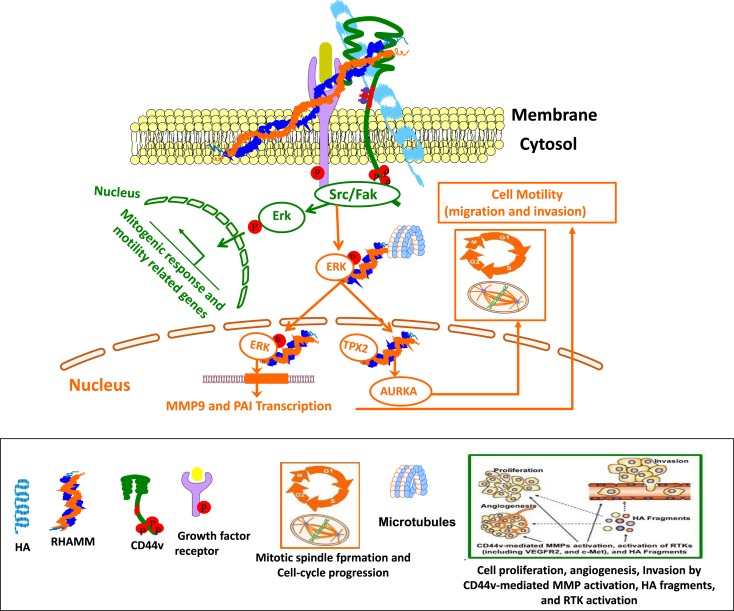
**Cross-talk between CD44 and RHAMM interaction with HA affects physiological and cellular functions**. Green Track is for extracellular RHAMM signaling involving CD44-HA-mediated pathways. Red track is for intracellular RHAMM signaling. Studies suggest a model for functions of CD44 and RHAMM. The interaction of HA-CD44-RHAMM affects physiological and cellular functions. Cell surface RHAMM interacts with CD44, HA, and growth factor receptors (GFR) to activate protein tyrosine kinase signaling cascades that activate the ERK1/2 MAP kinase cascade in a c-Src/Raf-1/MEK-1/ERK1/2 dependent manner (depicted in green track). In the absence of intracellular RHAMM, this signaling can stimulate the transcription of motogenic effectors to regulate a mitogenic response (cell proliferation/random motility). In the presence of intracellular RHAMM (red track), MEK-1/p-Erk1/2 also binds to a number of protein partners that allows activated RHAMM to enter the nucleus to regulate functions of microtubule dynamics via centrosome structure/function, and cell cycle progression via AURKA, a targeting protein for XkIp2 (TPX2). Activated RHAMM also controls expression of genes involved in cell motility such as PAI-1 and MMP-9.

Intracellular RHAMM binds to both actin filaments and microtubules in the cytoskeleton, in addition to interacting with ERK and SRC kinases (30, 268). Moreover, intracellular RHAMM also binds to a number of proteins, which can regulate microtubule dynamics and centrosome structure/function through ERK1/2/MAP kinase activation that contributes to microtubule-mediated cell polarity and cell migration. Nuclear RHAMM also binds to ERK1/2/MAP kinase, which mediates activation of PAI-1 and MMP-9 that are involved in cell motility and inflammation (132) (Figure 5). Secreted RHAMM can bind HA and, in concert with CD44, augment invasiveness in breast cancer. These interactions suggest that RHAMM may be necessary for CD44-mediated migration during inflammation, wound healing, tumorigenesis, and regulation of stemness/EMT phenotypes within tumor-initiating populations (256, 269, 270). Intracellular RHAMM, both cytoplasmic and nuclear, interacts with several signaling proteins and cytoskeletal components, including SRC, ERK1, actin, and microtubules (28, 29, 271). RHAMM regulates mitotic organization of microtubules through cytoskeletal elements aurora kinase A (AURKA), and BRCA1 (267, 272, 273), which is crucial for cellular fates, such as luminal differentiation and EMT. In addition, disruption of AURKA (274), BRCA1 (275), or RHAMM (276) modifies neurite extension, an alternate differentiation program dependent on microtubule nucleation (267) (Figure 5).

## Biology of HA-CD44/RHAMM Interaction

Given the crucial role that HA has in cancer cell survival, proliferation, and invasion, it is clear that HA interactions with both CD44 and RHAMM can induce numerous cell behaviors, including activation of tyrosine kinases, protein kinase C, FAK, and PI3K, MAPK, NFκB, and RAS, as well as cytoskeletal components required for inflammation and cancer (6, 24, 28, 132, 277) (model in Figure 5). While CD44 and RHAMM can interact independently with HA to induce cell behavior, in some cases their relative contributions result in redundant and overlapping functions. For example, in a collagen-induced model of arthritis, inhibiting CD44 function attenuates the disease, indicating the involvement of CD44 (193). RHAMM is significantly elevated in the knee synovial tissue of patients with advanced osteoarthritis compared to those without (278). These results are consistent with animal model studies demonstrating an isoform-specific role for RHAMM in collagen-induced arthritis (193). The findings from Nedvetzki et al. (193) indicate that arthritis in WT mice is CD44 dependent, whereas in CD44-knockout mice, it is RHAMM-dependent, as determined by antibody blocking and soluble peptide competition studies. This suggests that CD44 and RHAMM coexist in WT mice, but cell surface CD44 functions are dominant. Cell surface CD44 may primarily influence the joint inflammatory cascade in WT mice by its ability to quantitatively compete with cell surface RHAMM for HA and/or by its ability to regulate the potency of RHAMM-mediated signaling that may or may not be HA dependent. Absence of CD44 exacerbates bleomycin-induced lung inflammation in CD44-null mice due to accumulation of extensive HA ECM (215). In light of Nedvetzki’s findings (193), the augmented lung inflammation/fibrosis in bleomycin-induced CD44-null mice (215) may be due to RHAMM (193). Further, HA accumulation was greater in the arthritic joints of CD44-deficient mice than in those of wild type mice where CD44 (but not RHAMM) promotes endocytosis of HA and its subsequent lysosomal digestion. The excess HA in CD44-null mice may contribute to prolonged signaling through RHAMM (28), which might lead to aggravation of the inflammatory lung fibrosis. Thus, the loss of the CD44 gene could be compensated with enhanced expression of RHAMM. Moreover, the loss of CD44 allows enhanced accumulation of the HA in ECM, with which both CD44 and RHAMM interact (193). In other words, this study indicates dual functions of CD44 in arthritis, one that causes disease initiation and another that limits disease severity, at least in part by reducing pathology promoting effects of RHAMM (193).

Similarly, in RHAMM null fibroblasts, migration is impaired due to reduced CD44 at the cell surface, and to impaired ERK1 activation and impaired wound healing (259). Interestingly, the dual functions of CD44 exist in cancer where tissue-specific expression of specific variant isoforms of CD44v appears to control progression of some cancers (6, 94, 95), and these isoforms regulate tumor-initiating cells in sub populations of cancer cells (149). Similarly, overexpression of RHAMM controls severity of carcinogenesis (e.g., breast cancer, CRC, multiple myeloma) (239, 255, 279), and loss of RHAMM regulates peripheral nerve sheath tumor progression (280), suggesting duality of RHAMM performance to maintain cell behavior. Studies also indicate that the oncogenic role of RHAMM can be limited through the activation of p53 (281). Further, RHAMM and CD44v isoforms co-existed in 65% of the cancer cases with another 23% having either RHAMM or CD44v expression. Recent studies demonstrated that RHAMM- and CD44-mediated cell adhesion and motility appear reciprocal rather than overlapping (282), whereas concurrent expression of CD44 and RHAMM genes might confer tumorigenicity of gastric cancer cells (283).

This section has reviewed evidence for secretion of intracellular regulators of mitosis and differentiation in the determination of cellular migration, tumorigenesis, and inflammation by overlapping and independent interaction of CD44 and RHAMM with HA (Figures 3–5). The presence and functions of these multifunctional HA-receptors have been examined both in diseased human tissues as well as in animal models of diseases. The modulation of these receptors may dramatically alter disease functions. The next section discusses the emerging cell-based strategies to target these molecules in inflammatory diseases, and particularly in cancer.

## Therapeutic Approaches to Interrupt HA Interaction with Receptors

The inexorable course of progressive inflammation is similar in both the inflammatory diseases and in cancers. The seminal properties of these inflammatory and cancer cells involve cell adhesion, proliferation, migration, and invasion, and the HA-CD44 interactions can regulate these processes. Due to the unavailability of specific inhibitors, the physiological functions of HA have been mostly deduced from the biological changes caused by HA-receptor antagonists that can block the HA-CD44, and HA-RHAMM interactions. Here, we describe the possibility for therapeutic approaches to interfere with HA-CD44-RHAMM interactions (Table 2).

**Table 2:** **HA-CD44-RHAMM function in inflammation and cancer**.

Disease model	Function of HA in inflammation	Experimental approach by targeting HA-binding motifs	Reference
Bleomycin-induced lung Inflammation in αSMA-HAS2^+^/CD44-null mice	Unremitting inflammation and death, and accumulation of both HMW and LMW HA	CD44 antibody (Ab)	(295)
Excisional wound skin injury model	Acute and chronic skin inflammation	12-mer Pep-1 peptide	(132, 297, 306, 307)
		P15-1 peptide	
Staphlococcal aureus colonization of incisional skin wounds	Increased accumulation of CXCL1, CXCL2 and neutrophils after skin injury	Pep-35	(304, 305)
Bleomycin-induced lung injury mice model	Inflammation and fibrosis in lung injury	RHAMM (HABP) and RHAMM-like Peptide A	(295)

**Disease model**	**Function of HA in cancer**	**Experimental approach by using HA-drug carriers**	**Reference**

Bladder cancer	G2–M arrest and apoptosis	HYAL-1-v1	(312, 316–318)
	Mitotic/oncogenic responses	HA (~10-12kDa)-PTX conjugated drugs	
EMT-6 tumor spheroids	Increasing the accessibility to the chemotherapeutic drugs	Bovine testicular or bacterial hyaluronidase	(314)
Pancreatic ductal adenocarcinoma	Reduction of metastasis	Soluble form of PH20	(60, 61)
Colon, breast, esophageal, ovarian prostate cancer	Mitotic/oncogenic characteristics	HA (~200 kDa)-Irrinotecan conjugated drugs	(320–322)
Bone disease in cancer	Mitotic/oncogenic characteristics	HA (~750 kDa)-5-FU, doxorubicin	(323–325)
Colon cancer	Mitotic/oncogenic characteristics	HMW HA-bisphosphonate	(326)
CD44 positive cancer	Mitotic/oncogenic characteristics	Nanoparticle delivery of chitosan-HMW HA	(328–330)
CD44 positive cancer	Mitotic/oncogenic characteristics	Lipid-HMWHA-mitomycin C (HA-LIP)	(332–336)
CD44 positive HNSCC	Mitotic/oncogenic characteristics	Micelles to deliver paclitaxol, doxorubicin, Salinomycin	(338–340)
B16F10 murine melanoma and lung carcinoma cells	Mitotic/oncogenic characteristics	HA-nanocarrier to deliver doxorubicin, epirubicin	(335, 341)
Melanoma and breast cancers	Mitotic/oncogenic characteristics	Paclitaxel, mitomycin C, and various nanoparticles	(316, 341, 343–350)

### Role of HA, and HA-receptors in drug conjugates

The success of HA as a carrier depends on the number of receptors available on the target cells and on the affinity between the homing ligand and the receptor. HA preparations have been approved by the Federal Drug Administration as a medical device.

#### HA-Preparations Used for Non-Inflammatory/Non-Tumorigenic Therapies

(i) A transdermal drug delivery of insulin using micro needles (MNs) fabricated with 15% HA containing insulin is used in diabetes patients (284) (Table 2);
(ii) Transcutaneous immunization (TCI) of tetanus toxoid (TT) and diphtheria toxoid (DT) uses mixtures of sodium HA separately with TT and DT to form the MNs (285–288);
(iii) Antibodies against TNF-α or IL-1β conjugated to HMW HA diffuse slowly thus providing a sustained delivery of the antibodies in the wound (84);
(iv) Intra-articular injections have been used for the treatment of osteoarthritis of the knee, although further studies are required to establish its efficacy (289, 290); and
(v) HA has been approved as a surgical aid in eye surgery (291) and to improve skin elasticity (292).

#### Targeting HA and HA-Receptors in Anti-Inflammatory Therapies Using HA-Binding Peptide

Recent investigations have taken advantage of the peptide-based probes to develop imaging agents to target HA-binding regions of HA-receptors (Table 2). HA, CD44, and RHAMM are known to regulate immunity during tissue repair, including innate immune cells such as macrophages, and fibrogenesis (26, 28, 65, 112, 116, 132, 192, 210, 215, 259, 293–300). Thus, it is likely that HA-binding peptides can alter the HA-binding capacity of HA-receptors and modify disease processes such as tissue fibrosis, wound infection, contact hypersensitivity, and melanoma metastases in experimental models (293, 301–304).

CD44 and HA have been targeted for anti-inflammatory therapies using HA-binding peptides derived from a M13 phage-display library. One of these, Pep-1, which is a specific 12-mer HA-binding peptide, prevents leukocyte adhesion to HA (304) and inhibits leukocyte recruitment during contact hypersensitivity (304, 305). Pep-1 inhibits the binding of cells expressing HA-receptors (e.g., CD44) to immobilized HA substrate as well as the binding of soluble HA to cells expressing such receptors, implying that Pep-1 and HA-receptors compete for binding to the same ligand in acute skin inflammation (58). The peptide can also inhibit interleukin-2 induced vascular leak syndrome in mice by reducing damage to the endothelium, although lymphocyte migration was not affected (305). Pep-1 also inhibited secretion of the proinflammatory chemokine MIP-2 from HA stimulated macrophages (62). Studies on therapeutical aspects are currently under investigation that include the inhibition of enzymes that cleave CD44 (306) and CD44 vaccination (307), which provides partial resistance to experimental autoimmune encephalomyelitis (307). To date, no therapies have yet been developed that focus on augmenting the function of CD44 in the resolution of inflammation.

Using BLAST and ClustalX2, database searches between RHAMM and microtubule binding domains in microtubule binding proteins revealed only a moderate sequence homology of 17–24% to the HA-binding domain of RHAMM (295). Among the peptides, P15-1 preferentially binds oHAs (<10 kDa) with a moderate affinity, and this peptide specifically mimics and blocks HA-RHAMM-induced FAK signaling, resulting in the healing of excisional wounds (132, 295). This peptide had no visible effect on incisional skin injury repair, which is consistent with genetic deletion of RHAMM, which affects excisional but not incisional skin injury (259, 295, 308). Pep-35, which encodes two RHAMM HA-binding sequences, also reduced *Staphylococcus aureus* colonization of incisional wounds (302) by increasing the number of neutrophils and their expression of CXCL1 and CXCL2 after injury (303). Short peptides of RHAMM [hydrazide group to bisphosphonate (HABP)] and RHAMM-like (peptide A) also reduced inflammation and fibrosis in lung injury models by reducing macrophage migration and accumulation, and by reducing hydroxyproline (collagen) content in the lungs of the bleomycin injury mouse model (293). Similarly, RHAMM HA-binding peptides inhibited arthritis formation in collagen-induced arthritis in mice (193). These studies suggest that blocking HA-RHAMM, or HA-CD44 interactions could have therapeutic benefits in inflammation and wound repair disease processes in light of the dual roles of CD44 in the inflammatory response as discussed above (202, 216, 309) in specific diseases.

#### HA-Drug Conjugates in Cancer

##### Enzymatic degradation based therapeutics of HA

Hyaluronidases are a class of enzymes that predominantly degrade HA (Table 2). Recently, Lokeshwar et al. have shown that the expression of HYAL-1-v1 in bladder cancer cells that express wild type HYAL-1 induces G2-M arrest and apoptosis (310). It has been shown that adhesion of monocytes to human coronary artery smooth muscle cells was also inhibited by bacterial HYAL (311). Similarly, commercial bovine testicular or bacterial HYAL act as an anti-adhesive compound on EMT-6 tumor spheroids (312), and HYAL disaggregated EMT-6 spheroids increased chemosensitivity to cyclophosphamide (312), and also improved the therapeutic effectiveness of these agents, i.e., by increasing the accessibility of solid tumors to the chemotherapeutic drugs. Unlike EMT-6 cells, HYALs have limitations as an anti-adhesive agent for other human tumors (313) and can have side effects that impact normal tissue functions.

Development of a recombinant soluble form of PH20 (60) has paved the way for drug delivery in otherwise non-penetrable pancreatic ductal adenocarcinomas where HA forms a formidable barrier in the tumor stroma. Intravenous administration of PEGPH20 restored normal interstitial fluid pressure in the tumor by increasing vessel diameter. A prospective, randomized, placebo control trial in KPC mice with combined enzymatic and gemcitabine treatment has shown 83% increase in median survival rate. 80% placebo control mice died vs. 29% of Gem+PEGPH20 treated mice, and significant reduction of metastatic tumor burden was observed with combined therapy (61).

##### HA backbone-based conjugated drugs in cancer

Hyaluronan conjugated drugs are more soluble in water than the drugs alone (Table 2). For instance, the antimitotic chemotherapeutic agent paclitaxel (PTX) has low water solubility. Upon conjugation to HA, water solubility of the prodrug HA-PTX, and of HYTAD1-p20 (a HA-PTX conjugate renamed as ONCOFID-P by the pharmaceutical company Fidia) significantly increased CD44 dependent cellular uptake *in vitro* and *in vivo* in cancer cells, including bladder carcinoma cells (314, 315). Luo and Prestwich coupled PTX-*N*-hydroxysuccinimide ester (PTX-NHS) with HA of molecular weight ~11 kDa (316). PTX release from the hydrogel film was evaluated *in vitro* using selected anti-bacterial and anti-inflammatory drugs (317). The pharmaceutical company Fidia prepared ONCOFID™-S, another HA prodrug conjugate with SN-38, the active CPT11 (irinotecan) metabolite. The HA used had a molecular weight of ~200 kDa. *In vitro* and *in vivo* phase I and phase II clinical studies were initiated using ONCOFID-S in several CD44-overexpressing cancer cells, including colon, gastric, breast, esophageal, ovarian, and human lung cancer cells. In all these studies, these drugs reduced tumor cell growth and metastasis (318–320).

##### HA-encapsulated drugs

Another strategy for HA-based CD44 targeting utilizes the concept that the large volume domain of HA (molecular weight > 750 kDa) can non-covalently entrap small therapeutic molecules within its domain (Table 2). HA was then used as a macromolecular carrier for the irinotecan drug along with its targeting properties (321). Clinical trials of three HA formulations [termed hyaluronic acid chemo transport technology (HyACT)] have been undertaken in Australia. Phase I clinical evaluation of two formulations based on HA (HyACT) with 5-fluorouracil (5-FU) (known as HyFIVETM), and on HA (HyACT) with doxorubicin (DOX) (known as HyDOXTM) demonstrated reasonable cytotoxic efficacy without compromising safety of these formulations (322, 323).

##### HA-tailed drug carriers

These include the following HA conjugates with cytotoxic activity (Table 2).

(i) Bisphosphonates (BPs) – where HMW HA is linked via a HABP (324).
(ii) Carbonates (HA-pCB) – where *n*-propyl carbonate is linked to HA via an ester linkage (325, 326).
(iii) Chitosan – where chitosan-HA nanoparticles (HA-CTNPs) containing 5-FU/oxaliplatin were prepared by the ionotropic gelation method. 5-FU/oxaliplatin loaded HA-CTNP formulation significantly enhanced cytotoxicity compared with either chitosan nanoparticles (CNTPs) alone or free 5-FU, or oxaliplatin in HT29 CRC cell lines, which overexpress CD44 (327, 328).
(iv) Gagomers (GAG-mers) – where GAG-mers (GAG cluster of particles) are composed of lipid molecules that self assemble into particulate clusters in hydrophilic solutions, which are then covalently coated with HMW (1.2–5 MDa) HA. When tested in primary head and neck cancers and normal cells taken from the same patient (329), GAG-mers selectively bound only to the tumor cells to induce cytotoxic activity.
(v) Liposomes/lipoplexes (HA-LIP) (330–334) – where HMW HA was decorated on nano-sized encapsulated mitomycin C (MMC). The cytotoxic activity of the drug loaded into HA-LIP was found to be ~100-fold that of free drug in *in vitro* and *in vivo* tumor cells overexpressing the HA-receptors, but not in cells with low receptor expression levels. The HA-LIP conjugate can be used to deliver plasmid DNA and small interfering RNA (siRNA) to CD44 positive cancer cells (332, 335). The presence of HMW HA in the lipoplexes enhanced nucleic acid protection from degradation by DNase I or RNAse VI. In case of LMW HA, the HA was linked to PE to form a conjugate in which only one PE molecule is linked to a HA molecule (333, 334). This procedure enables a controlled amount of HA to be introduced into the liposomes. oHAs were attached to PE and incorporated into the liposomes, which increased their recognition, cytotoxicity, and transfection efficiency by tumor cells expressing high levels of CD44 in a temperature-dependent manner.
(vi) Micelles – where the hydrophilic backbone of HA was conjugated via its carboxyl groups to amino functions of poly-l-histidine (PHis) or polyethylene glycol (PEG). These HA constructs form nanocomplexes by self-organizing into micelles, and they can carry anticancer drugs, including PTX. In addition, PTX when entrapped into the hydrophobic cores of the folic acid (FA)-conjugated HA-C18 micelles exhibited higher cytotoxic activity compared to Taxol in MCF-7 cells that overexpress both the folate receptor and CD44. The micelles of HA-PTX (336), HA-DOX (337), and HA-salinomycin (338) exhibited more pronounced cytotoxic effects on HA-receptor overexpressing cancer cells than on receptor deficient cells.
(vii) Nanocarrier – where HA was conjugated to a nanocarrier. These nanoparticles were able to deliver anticancer drugs, including epirubicin (339), DOX (333), PTX (314), and MMC (339), as well as siRNA, to CD44 overexpressing cells (340). In addition to the well-developed strategies described above, several multifunctional nanocompounds have recently been developed that combine therapeutic and diagnostic properties. These nanoparticles include quantum dots (341), carbon nanotubes (342) and nanodots (343), graphene (344), gold nanoparticles (345), iron oxide nanoparticles (346), and silica nanoparticles (347), and they have been found to acquire novel characteristics after their conjugation with HA (341–348) (Table 2).

### Targeting CD44 in cancer

HMW HA can interact with a number of CD44 receptors and be endocytosed. It is rapidly cleared from circulation by the liver hepatocytes (349), and any excess of the targeting compound can lead to adverse effects (350). This rapid clearance was circumvented by choosing oHAs long enough to bind to CD44 but too short to bind to the HARE receptor, which may permit targeting to cells that overexpress CD44. The minimum HA length required to interact with individual CD44 molecules is 6–10 monosaccharides (220) with moderate affinity.

#### Interrupting HA-CD44 Interaction

**Figure 6:**
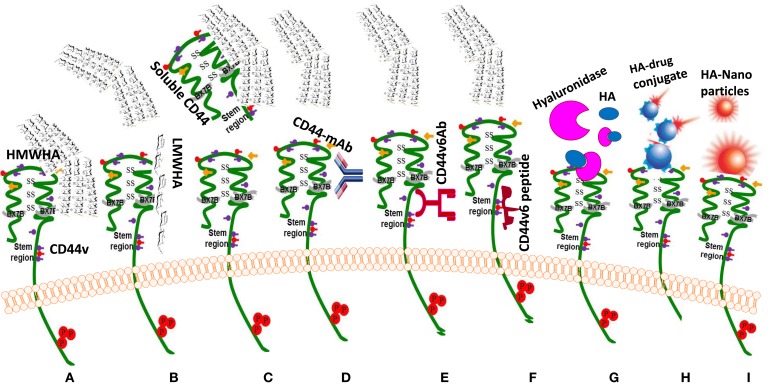
**Drug targeting approaches to exploit HA-CD44 interaction (C) in cancer treatment**. **(A)** HMWHA-CD44v interaction induces inflammation and tumor growth; most common blocking reagents against CD44 isoforms: **(B)** LMW HA inhibits the binding of HMWHA; **(C)** sol-CD44 competes with HMWHA to bind with CD44v; **(D)**. CD44-blocking monoclonal antibody (CD44-mAb); **(E)** antibodies against CD44v6; **(F)** peptides blocking binding of HA with CD44v6; **(G)** enzymatic hydrolysis by hyaluronidase cleaves the HMWHA to small fragments and blocks the HA-CD44v6 interaction; **(H)** targeting HA receptor with HA-drug conjugate; **(I**) targeting “HA receptor” with “HA nanoparticles.”

As mentioned in the previous sections, the main property attributed to CD44 is its ability to bind HA, and this binding contributes to apoptosis resistance of cancer-initiating cells (6, 37, 351–353) (Table 3; Figure 6). There are also examples in which CD44 cross-linking initiates apoptosis. However, apoptosis induction by HA-CD44 cross-linking is largely restricted to non-transformed cells with immature leukocytes being most easily affected. Thus, together with the oncogenic transformation and the pronounced association of CD44 with oncogenes, HA-CD44 cross-linking initiates signals that promote cell survival in tumor cells. The cross-talk of CD44 with multidrug resistance genes accounts for an alternative CD44-mediated apoptosis resistance mechanism This approach involves substituting multivalent interaction of HMW HA with CD44 with monovalent interaction of small oHAs [6–18 saccharide units (oHAs)] (217, 354). The oHAs inhibit HA-CD44 downstream cell survival and proliferation pathways, and they stimulate apoptosis and expression of phosphatase and tensin homolog (PTEN) (86, 355). The oHAs also sensitize cultured cancer cells to some chemotherapeutic drugs by inhibiting expression of MDR1 and other ABC transporters (96, 228, 241). While oHAs inhibit the growth of several tumors implanted as xenografts (86), they did not give consistent significant growth inhibition in adenoma growth in Apc Min/+ mice (Misra et al., unpublished results). There are several studies contradicting the cellular response of HMW and LMW HA. For example, in schwannomas cells, HMW HA inhibited tumor growth (356). In contrast, LMW HA can induce angiogenesis (80) and inflammatory responses in various cell types (76, 112, 357). These inconsistencies could be resolved if the HA preparations and size determinations could be done properly and if the receptors, or the signaling pathways were identified properly. Only a few studies define the receptors (93, 232). Thus, we developed siRNA and, even more advantageous, short hairpin RNA (shRNA), to target CD44v6 in colon cancer, and showed that they can successfully interrupt HA-CD44v6 interaction and signaling (~90-95%). We then developed a novel shRNA delivery approach to target HA-CD44v6 specifically in tumor cells (6, 94, 95, 248), which is discussed in the following sections.

**Table 3:** **CD44 function in cancer**.

Disease model	Function of CD44 in cancer	Approaches to interfere the HA-CD44 interaction	Reference
Colon, breast and prostate cancer cells	Apoptotic resistance	HA oligosaccharides [6–18 saccharide units (oHAs)]	(86, 96, 219, 230, 243, 356, 357)
Schwannomas cells	Inhibition of cell survival	HMW HA	(358)
Xenografts of mammary and colon tumor cells	Cell survival	Soluble CD44	(166, 230, 231, 355, 360, 361)
HNSCC	*In vitro* and *in vivo* malignant properties	Anti-CD44-Mo-Ab conjugated with mertansine, or radionucleotide, activated anti-CD44 antibody (H90)	(152, 369–373)
AML cells	Tumor cell survival activities	CD44v6 peptides containing the v6 exon region	(373)
Colon cancer cells	Tumor cell survival activities	Penetratin-conjugated peptide, and peptide specific for CD44	(103, 374, 375)
Melanoma and prostate tumor cells	*In vitro* cell migration, invasion	Pep-1 (specific 12-mer HA-binding peptide	(379)
Melanoma tumor cells	Melanoma tumor cells growing under both anchorage-dependent and -independent conditions melanoma tumor growth *in* nude mice xenograft models	BHP (42 amino acid peptide containing three BX7B HA-binding motifs) peptide	(362)

**Disease model**	**Function of CD44 in cancer**	**Systemic targeting by viral and non-viral vectors**	**Reference**

Ornithine transcarbamylase deficient sparse fur mice	Tumor cell growth *in vitro* and *in vivo*	Viral vectors	(389)
K562 cells	Tumor cell growth *in vitro* and *in vivo*	Non-viral vectors	(390, 391)
Intestinal tumors in Apc Min/+ mice	Tumor cell growth *in vitro* and *in vivo*	Tissue-specific delivery of non-viral CD44v6 shRNA-nanaoparticle	(94)

Overexpression of sol-CD44 displaces exogenous HA from its binding to CD44 and thereby retards tumor cell growth both *in vitro* and *in vivo*, and sensitizes tumor cells to chemotherapeutic drugs (228, 229, 353, 358, 359). However, soluble forms of CD44 can differ in their capacity to bind HA, indicating that sol-CD44 can act as a competitive inhibitor of exogenous HA-protein interactions. In these studies, sol-CD44 blocks HA-activated CD44 clustering and inhibits CD44-mediated recruitment of activated MMP-9 and invasion potential of cancer cells (165). Similar to HA-binding sol-CD44, a peptide (42 amino acids in length) enriched with three HA-binding motifs (BX7B) possesses an antitumor activity in melanoma cancer (360).

#### Targeting with Anti-CD44 Antibodies

Anti-CD44 antibodies against highly expressed CD44v variants can effectively target drugs to cells expressing a selective CD44v, which can then inhibit and disrupt CD44 matrix interactions, alter CD44 signaling, and cause apoptosis (361) (Table 3; Figure 6). Antibodies against highly expressed variants can also be designed to selectively deliver a cytotoxic drug to cancer cells in two different ways. They can bind and neutralize the receptor by competitive inhibition of its ligand and hence stop the receptor-signaling cascade. They can also be attached to radioisotopes, toxins, or chemotherapeutic agents and target them to the required vicinity. The first concept has been effectively utilized for other receptors like anti-EGFR antibodies cetuximab, panitumumab, etc. Anti-CD44v6 conjugated with a cytotoxic drug mertansine has been extensively studied in early phase clinical trials for its value in translational medicine (362–366). Head and neck squamous cell cancer (HNSCC) patients were treated with three doses of mertansine conjugated bivatuzumab (humanized anti-CD44v6 monoclonal antibody [HAMA] labeled with technetium-99m) was first tested (151, 367, 368). However, the phase I clinical trials were rather disappointing in terms of accumulation and toxicity. Furthermore, dose limiting skin toxicity was observed, most probably due to CD44v6 expression in non-tumor skin areas, indicating limitations in the use of this antibody therapy. However, given the promising results of a phase I clinical study with the radionuclide-antibody conjugates (362–366), new similar conjugates of bivatuzumab with radioisotopes were found to be safe and effective in phase I trials (369, 370), and a conjugate of immunotoxin with bivatuzumab (369) was also found to be safe in the next clinical trial on 30 HNSCC patients. In this context, a previous study showed that activated anti-CD44 antibody (H90), when used in human acute myeloid leukemia (AML) cells, reduced the leukemic repopulation by altering the fate of AML leukocyte stem cells (AML LSC), and by abrogating AML LSC homing, leading to their death (371). This study suggests that activated anti-CD44 antibodies without conjugation with toxin are much safer and efficient for antitumor activity. Thus, CD44, particularly CD44 variant forms, remains a crucial target for tumor therapy (Figures 4 and 5). To address this issue, we have developed a novel tissue-specific (CD44v6) shRNA delivery strategy by a Cre-lox system. This technology is discussed in a following section.

#### Peptide-Based Strategies

It is becoming increasingly evident that future prospects for the treatment of inflammation/cancer should include the targeting of specific signaling pathways in tumor cells. (Table 3; Figure 6) CD44v6 is a co-receptor for VEGF/VEGFR-2 and HGF/c-Met, and mutational analysis of CD44v6 revealed that three amino acids in the v6 region are required for its co-receptor function for Met and VEGFR-2. These studies helped to identify CD44v6 peptides with the minimal length of five amino acids that contain the critical v6 exon region that can inhibit both VEGFR-2 and c-Met activation. This inhibits the co-receptor function of CD44v6 and the vascularization and tumor cell growth, migration and invasion (103, 372).

Serine phosphorylation of the human CD44 cytoplasmic tail at Ser323 and Ser325 enhances cell migration potency. Thus, a penetratin-conjugated peptide containing phosphoserine at residue 325 reduced *in vitro* migration of melanoma cells (373). Insertion of phosphorylated Ser325 (pSer325) or pSer323 and pSer325 in the peptides disrupted the activation of CD44/MMP-9 signaling complex in prostate cancer cells (374). Another peptide comprising eight amino acids that bind specifically to CD44 (375) and derived from human urokinase plasminogen activator (A6) inhibits migration, invasion, and metastasis of cancer cells by interfering with an uPA-independent signaling pathway (376).

The Pep-1 (specific 12-mer HA-binding peptide) peptide can reduce lung metastasis and prolong survival of mice injected with cell line-derived melanoma cells (377). Besides this Pep-1, BH-P, a 42 amino acid peptide containing three BX7B HA-binding motifs present in CD44 can exert antitumor effects by inhibiting the proliferation of melanoma tumor cells growing under both anchorage-dependent and -independent conditions and by inhibiting melanoma tumor growth in nude mice xenograft models (360).

#### Tissue-Specific Deletion of CD44variant Signaling

CD44 splicing can regulate interaction with HA (378, 379) (Table 3; Figure 6). HA is not only the essential component of the tumor matrix assembly (380), but also has a crucial role in cancer stem cell (CSC) niches, which are particularly rich in HA (381). Interference in matrix components alters signaling events of tumor-initiating cells such that tumor cells that express HA can induce expression of HA in other cells that make up the CSC niche (382, 383). In addition, CD44v6 isoforms are engaged in matrix assembly (384) and have been identified as markers of CSCs in colon cancer, and they account for the metastatic susceptibility of the tumors (385). In the intestinal mucosa, CD44 is a major direct target of β-catenin mediated transcription (177), and we have shown that CD44v6 also regulates β-catenin in colon cancer cells (93). Furthermore, CD44v4-v10, but not CD44s, is a crucial component of the intestinal stem cells in the crypts of ApcMin/+ mice, and controls tumor initiation and relapse by controlling the balance between cell survival and apoptosis (178). These studies indicate that CD44v6 targeting in colon cancer is a promising therapeutic approach. The inhibition of CD44 mRNA expression by inducing the expression of siRNA/shRNA in tumor cells is an alternative approach to the use of CD44-blocking antibodies to interfere with the function of CD44 proteins. This section discusses the fundamental aspects of a therapeutic approach targeting CD44v6 by means of colon cancer cell-specific delivery of shRNA. This approach addresses: (a) what to deliver (engineered therapeutic CD44v6 shRNA), (b) how to deliver (delivery strategies using non-viral transferrin (Tf)-coated PEG-polyetheleneimine (PEI) (Tf-PEG-PEI) nanoparticles for *in situ* cell-specific therapy), and (c) where to deliver (tumor cell targets, in particular, colon tumor cells for *in situ* cell-specific therapy). The technique of using shRNA in an expression vector is an alternative strategy to stably suppress selected gene expression, which suggests that the use of shRNA expression vectors holds potential promise for therapeutic approaches for silencing disease causing genes (386). There are two ways to deliver shRNA in cancer cells, either using a viral vector or a non-viral vector. Viral vectors have been used to achieve proof of principle in animal models and, in selected cases, in human clinical trials (387). Systemic targeting by viral vectors toward the desired tissue is difficult because the host immune responses activate viral clearance. Systemic administration of a large amount of adenovirus (e.g., into the liver) can also be a serious health hazard and even caused the death of one patient (387). Nevertheless, there has been considerable interest in developing non-viral vectors for gene therapy.

**Figure 7:**
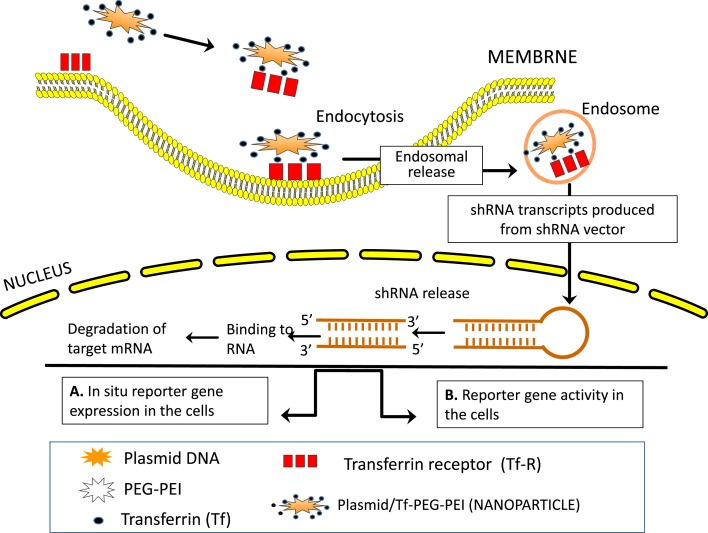
**Schematic illustration of cellular uptake of plasmid DNA/Tf-PEG-PEI (nanoparticles) polyplexes, their shielding from non-specific interaction, and the mechanism of action of shRNA**. Internalization of PEG-shielded and [transferrin receptor (Tf-R)]-targeted polyplexes into target cells occurs by receptor-mediated endocytosis after association of polyplex ligand Tf to Tf-R present on the target cell plasma membrane. Internalized particles are trafficked to endosomes followed by endosomal release of the particles and/or the nucleic acid into cytoplasm. Released siRNA will form a RNA-induced silencing complex and will be guided for cleavage of complementary target mRNA in the cytoplasm. SiRNA (antisense) guide strand will direct the targeted RNAs to be cleaved by RNA endonuclease. Finally, plasmid/Tf-PEG-PEI-nanoparticles delivery in the target cell shows reporter gene expression and activity. The normal tissue cells are not affected because they do not make the targeted CD44 variant. Tf-PEG-PEI nanoparticle coated plasmids (pSico-CD44v6 shRNA/pFabpl-Cre) circulating in blood accumulate at tumor regions enhanced by the EPR effect. Endocytosis mediated by ligand-receptor interactions occurs because the nanoparticles are coated with the Tf-ligand for the Tf-R receptor on the tumor cell surface.

**Figure 8:**
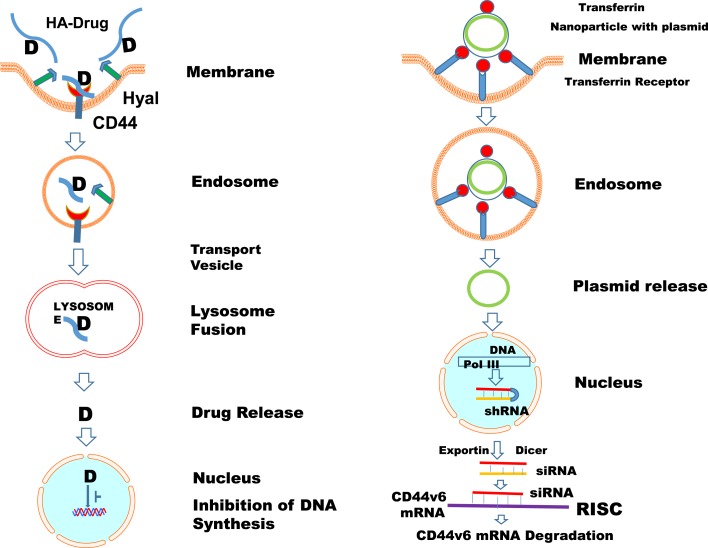
**Exploitation of HA-CD44 interaction for anticancer therapy**. Left panel shows a schematic diagram for cancer cell internalization of HA conjugated to a drug inhibitor of DNA synthesis. CD44 on the membrane traps the HA-drug conjugate and efficiently internalizes it by endocytosis and forms an endosomal vesicle. This is then transported to the lysosome and fused, and the internalized HA is degraded by hyaluronidase 1 (Hyal-1) into small HA oligosaccharides and then to monosaccharides by lysosomal glycosidases, which ultimately releases the conjugated drug. The drug then goes to the nucleus and inhibits the DNA synthesis. Right panel shows targeting the CD44v6mRNA in cancer cells by CD44v6 shRNA. Plasmids that produce CD44v6 shRNA are coated with transferrin present on the outer surface of the nanoparticles. The transferrin molecules then target the transferrin receptors present in high amounts on the cancer cells. Upon internalization, an endosome forms and the plasmids are released to the nucleus where CD44v6 shRNA is produced by DNA pol III. The shRNA is then exported out of the nucleus by exportin, and the dicer enzyme converts shRNA into CD44v6 siRNA. One of the strands of siRNA will bind to CD44v6 mRNA and form a RISC (RNA-induced silencing complex) that is ultimately degraded. Adapted from our article in the International Journal of Cell Biology.

Figures 6–8 illustrate the model for the uptake of non-viral vectors through Tf-PEG-PEI-nanoparticles carrying multiple functional domains. Non-viral vectors mediate unspecific interactions with non-target cells and blood components, which results in the rapid clearance from circulation. PEI has positive charges and binds to negatively charged plasmid DNA to form condensed particles. The PEG shields the condensed PEI-Plasmid particles from unwanted interactions and prevents clearance from circulation thus giving longer half-life (388). To increase the transfection efficiency of the shielded particles (plasmid DNA/PEG-PEI), different targeting ligands, such as peptides, growth factors and proteins, or antibodies, have been incorporated into the vectors (389). One such targeting ligand is Tf, an iron-transporting protein that is recognized by Tf receptors (Tf-Rs). Association of Tf to polyplexes enhances transfection efficiency (389). This concept was tested by preparing non-viral vector Tf-PEG-PEI-nanoparticles with plasmids packed inside an outer PEG-PEI layer coated with Tf that binds with Tf-R with high affinity in the tumor cells (94, 389, 390) (depicted in the model in Figure 7). We found that the Tf-R is present at much higher levels on the tumor cells than on phenotypically normal epithelial cells (94). Tf-PEG-PEI-nanoparticles significantly enhance transfection efficiency of CD44v6 shRNA generator plasmids by promoting the internalization of the nanoparticles in proliferating and non-proliferating colon cells through receptor-mediated endocytosis (94, 389). Therefore, the uptake of Tf-PEG-PEI-nanoparticles carrying multiple functional domains (surface shielding particles Tf-PEG-PEI, CD44v6 shRNA generator plasmids, tissue-specific promoter driven Cre recombinase plasmids, and conditionally silenced plasmids) can overcome the intracellular barriers for successful delivery of the CD44v6 shRNA (94).

This CD44v6 shRNA plasmid delivery approach was tested for transfection of pSV-β-gal/Tf-PEG-PEI-nanoparticles in cellular models (6, 94, 248) (Figure 7). Following this experiment, we successfully demonstrated that the CD44v6 shRNA is localized into the colon tumor cells by an end point assay of CD44v6 expression and by perturbation of HA-CD44v6 interaction as reflected in the reduction in the number of tumors (94). The tissue-specific shRNA delivery was made possible by the use of Cre-recombinase produced in response to a colon tissue-specific promoter, which deletes the interruption between the U6 promoter and the CD44v6 shRNA oligonucleotide. The newly developed cell-specific shRNA delivery approach by Misra et al. (94) confirmed that targeting the signaling pathways induced by HA-CD44v6 interaction inhibited distant colon tumor growth in Apc Min/+ mice. Our recent unpublished *in vivo* studies with the C57Bl/6 mice have now shown that systemic delivery of a mixture of two plasmids, prostate-specific Probasin-Cre/Tf-PEG-PEI-nanoparticles and floxed pSico-CD44v9shRNA/Tf-PEG-PEI-nanoparticles, can target both localized and metastatic prostate cancer cells. This novel approach opens up new ways to combat cancer and to understand tumorigenesis *in vivo* for the following reasons (Figure 7).

(i) Cell-specific shRNA to CD44variant (CD44vshRNA) is released by applying a tissue-specific promoter driven Cre-lox mechanism.
(ii) This shRNA silences the expression of the selected CD44 variant in the target tissue cancer cells.
(iii) This shRNA does not affect the normal target tissue cells, which rely on the standard CD44s and do not express the targeted CD44variant, and therefore are not affected by the plasmids.
(iv) The target CD44vshRNA will not be expressed in other types of cells because the tissue-specific promoter only unlocks the Cre-recombinase in the targeted tissue cells, which reduces potential side effects (94).
(v) The Tf-PEG-PEI-nanoparticles that carry plasmids are biodegradable and cleared from the system.
(vi) This method inhibits the pathophysiological role of HA-CD44v interactions in cancer.
(vii) It can establish diagnostic markers for the targeted cancer, including CD44variants, soluble CD44, and HA.
(viii) It can identify HA-CD44v interactions as innovative novel therapeutic targets against cancer progression.

Thus, the conditional suppression of gene expression by the use of a CD44vshRNA expressing plasmid holds potential promise for therapeutic approaches for silencing HA-CD44variant signaling and downstream signaling pathways that promote disease causing genes (386).

### Targeting RHAMM in cancer

Under homeostatic condition, RHAMM expression is very low. Its expression is increased during pathogenesis (inflammation and cancer) (132). Such a restricted expression of RHAMM makes it a suitable target for cancer and wound repair therapy with low toxicity (132).

#### RHAMM-Based Immunotherapies

Peptides that mimic either RHAMM itself or the HA sizes that bind to RHAMM have been shown to affect neoplastic processes as well as inflammation and wound repair (Table 4). HA targeting by Pep-1 blocked esophageal squamous cell carcinoma invasiveness by inhibiting HA-RHAMM induced effects (391). There is evidence that RHAMM-R3 peptides that are currently being tested in phase II clinical trials for multiple myeloma and myelodysplastic syndrome show efficacy and low toxicity in patients (392, 393). RHAMM silencing abrogated the self-renewing property of glioblastoma stem cells, and loss of RHAMM in malignant peripheral nerve sheath tumors or multiple myeloma sensitizes tumor cells to inhibitors (394–396). Neuroblastoma tumors are also sensitive to RHAMM-based immunotherapies due to the established roles of AURKA (397) and BARD1 [breast cancer gene 1 (BRCA1)-associated ring domain protein-1] (398), two components of the AURKA-BRCA1/BARD1-RHAMM-TPX2 centrosome module, within this refractory disease (267).

**Table 4:** **RHAMM function in cancer**.

Disease model	Function of RHAMM in cancer	Approach using HA-binding peptide	Reference
Esophageal squamous cell carcinoma	Invasiveness	Pep-1	(393)
Multiple myeloma and myelodysplasia syndrome	Anti-apoptosis	RHAMM-R3 peptides	(394, 395)
Malignant peripheral nerve sheath tumors or multiple myeloma	Apoptotic resistance	RHAMM silencing	(396–398)
Neuroblastoma tumors	Apoptotic resistance	RHAMM-based immunotherapies targeting AURKA and BARD1	(399, 400)
Acute myeloid leukemia	Antitumor activity	RHAMM R3	(395, 401)

#### Cell-Based Therapies

In addition, cell-based strategies using peptide vaccination with a RHAMM-derived, highly immunogenic peptide, termed RHAMM R3, has proven safe and effective at generating CD8+ RHAMM-specific T cell cytotoxic cellular responses and antitumor activity in patients with AML, myelodysplastic syndrome, multiple myeloma, and, more recently, chronic lymphocytic leukemia in phase I/II trials (393, 399) (Table 4). These studies provide evidence that blocking RHAMM is a promising immunotherapy approach in patients with hematological malignancies. In another study, antitumor activity has also been reported for vaccination with dendritic cells expressing exogenous RHAMM mRNA in a mouse model of glioma (400).

A handful of RHAMM-based therapeutical studies in cancer suggest that more intense studies should be undertaken to determine how RHAMM signaling contributes to cancer progression. Although HA-receptor-mediated signaling is believed to be a promising anticancer/anti-inflammatory drug target, given the dual functions of HA, CD44 and RHAMM in the inflammatory/tumorigenic responses, caution must be taken in considering therapeutic targeting of these molecules, which should be designed for specific organs and specific diseases with consideration of the use of proper sizes of endotoxin free HA, which might provide a way to prevent side effects.

## Conclusion

In the past few years, basic and clinical research on CD44 have identified the genomic DNA structure and alternative splicing pattern of CD44, which has led to a conclusion that CD44v is not one but a family of proteins, and that discrete isoforms are expressed and regulated at various stages of oncogenic transformation. Most, but not all, cancers overexpress discrete species of CD44v, which can be correlated with tumor aggressiveness. A challenging area of research would be to define what cellular functions are associated with the various CD44 isoforms that are overexpressed in various cancers before a CD44-based therapy can be undertaken. Considering the biological functions of HA particularly the connection of such function with molecular size of HA, the HA-binding proteins, its spatial and temporal distribution in tissues, and the cellular background and tissue stages, care must be taken to ensure the long-term safety of HA-based bioconjugates. Thus, this review defines the origins of evidence for a linkage between HA, CD44v, and RHAMM expression with inflammatory diseases, including malignancy, and emphasizes the most advanced and developed therapeutic strategies, those that have either been used for clinical trial or are nearly ready to get there. In addition, approaches used in various preclinical models are also briefly reviewed. Studies reviewed here identified strong prospects for anti-CD44 therapies. The HA-CD44 interaction system is illustrated in Figure 8 where we specify cancer therapeutical aspects (discussed in this review) that specifically perturb HA-CD44 signaling pathways. Interference with the function of HA-CD44 can inhibit the inflammation/malignant processes at multiple stages. This can be accomplished by perturbing HA-CD44 signaling pathways, by disruption of the HA matrix with HYALs to facilitate passive carrier uptake and providing a sustained source of drug at the tumor site, by targeting CD44 with a CD44-blocking antibody, or by tissue-specific targeting of specific variants of CD44v that are overexpressed in tumors (Figure 8).

Although overexpression of CD44 correlates with bad prognosis in patients with most human cancers (151, 178–184), it was also found that CD44 is extremely sensitive to changes in the microenvironment. For example, CD44 in breast cancer cells, neuroblastomas and prostate cancer may act as a metastatic suppressor gene (170, 185, 186), suggesting that the growth promoting pathways in these tumors are independent of CD44. These differential regulations should be considered carefully while designing CD44 as a target for therapeutic strategy.

For last two decades, several studies were dedicated to the use of CD44v, in particular CD44v6, as a therapeutic target. As discussed in Section “Targeting with Anti-CD44 Antibodies,” the anti-CD44v6 antibody conjugated with mertansine showed dose limiting skin toxicity due to CD44v6 expression in non-tumor skin tissue. However, the scientific progress in the last few years provides strong support for using CD44v as a target for therapeutic strategies. This is due to the fact that: (1) CD44v6 is a marker for colon CSCs (180, 385); (2) CD44v6 can act as co-receptor for at least three RTKs (c-Met, VEGFR-2, and EGFR) (103, 236, 401), and many of the oncogenic functions of CD44v6 can be attributed to downstream signaling induced by these RTKs; and (3) CD44v6 is highly expressed in many cancers. Nevertheless, there is an urgent need to define which CD44v variants are present on the CSCs for the particular type of cancer, and then target them in signaling complex tissue specifically by non-viral vectors. Collectively, these studies suggest that future development of drug targeting approaches can use tissue-specific expression of CD44v-specific antagonists as well as inhibitors (agents that are currently used in the clinic) targeting CD44 isoforms and their co-receptors/ligands that alter intracellular signaling in the inflammatory/tumor tissue microenvironment, as an effective and novel approach to regulate these diseases. Finally, since CD44 and RHAMM bind to HA, targeting against RHAMM may be an additional treatment option.

## Author Contributions

SG and SM wrote the review. Dr. VH has reviewed the draft and final version, and the revised draft of the manuscript. Dr. RM has edited the final and revised version of the manuscript.

## Conflict of Interest Statement

The authors declare that the research was conducted in the absence of any commercial or financial relationships that could be construed as a potential conflict of interest.

## Abbreviations

CD44v, variant isoform of CD44; GAG, glycosaminoglycan; HA, hyaluronic; HAS, hyaluronan synthase; HMW HA, high molecular weight HA; HYAL, hyaluronidase; LMW HA, low molecular weight HA; oHAs, HA oligosachharides; PEG, polyethylene glycol; PEI, polyethyleneimine; PTX, paclitaxel; RHAMM, receptor of HA-mediated motility; RTK, receptor tyrosine kinase; shRNA, short hairpin RNA; siRNA, short interfering RNA; Tf, transferrin.
